# Establishing national hospital costing systems: insights from the qualitative assessment of cost surveillance pilot in Indian hospitals

**DOI:** 10.1136/bmjopen-2023-082965

**Published:** 2024-09-10

**Authors:** Yashika Chugh, Shuchita Sharma, Lorna Guinness, Deepshikha Sharma, Basant Garg, Abha Mehndiratta, Shankar Prinja

**Affiliations:** 1 Community Medicine and School of Public Health, Post Graduate Institute of Medical Education and Research, Chandigarh, Chandigarh, India; 2 Department of Community Medicine, Post Graduate Institute of Medical Education and Research, Chandigarh, Chandigarh, India; 3 Center for Global Development, Europe, London, UK; 4 London School of Hygiene and Tropical Medicine, London, UK; 5 National Health Authority, Government of India, New Delhi, India

**Keywords:** PUBLIC HEALTH, Health policy, Health Workforce

## Abstract

**Abstract:**

**Objective:**

The Indian Government launched Ayushman Bharat Pradhan Mantri Jan Arogya Yojana (PM-JAY), the world’s largest health insurance scheme, in 2018. To reform pricing and gather evidence on healthcare costs, a hospital cost-surveillance pilot was initiated among PM-JAY empanelled hospitals. We analysed the process and challenges from both healthcare providers and payer agency’s perspectives and offer recommendations for implementing similar systems in lower- and middle-income countries.

**Design:**

We employed an open-ended, descriptive and qualitative study design using in-depth interviews (IDI) as the data collection strategy.

**Settings:**

The interviews were conducted in both virtual and face-to-face modes depending on the convenience of the participants. The IDIs for the National Health Authority (NHA) officials and all providers in Kerala were conducted virtually, while face-to-face interviews were conducted and in Haryana and Chhattisgarh.

**Participants:**

Staff from 21 hospitals in three states (Haryana, Chhattisgarh and Kerala), including officials from State Health Agency (n=5) and NHA (n=3) were interviewed.

**Results:**

The findings highlight significant challenges in reporting cost data at the hospital level. These include a shortage of trained staff, leading to difficulties in collecting comprehensive and high-quality data. Additionally, the data collection process is resource-intensive and time-consuming, putting strain on limited capacity. Operational issues with transaction management system, such as speed, user-friendliness and frequent page expirations, also pose obstacles. Finally, current patient records data has gaps, in terms of quantity and quality, to be directly put to use for pricing.

**Conclusion:**

Accurate cost data is vital for health policy decisions. Capacity building across healthcare levels is needed for precise cost collection. Integration into digital infrastructure is key to avoid burdening providers and ensure quality data capture.

**STRENGTHS AND LIMITATIONS OF THIS STUDY**

- A comprehensive process evaluation of the healthcare cost surveillance pilot from the perspective of both providers and payer agencies.
- The characteristics of the providers interviewed included public, private and trust hospitals with various bed capacities and accreditations by the National Accreditation Board for Hospitals & Healthcare Providers to ensure representativeness.
- The interviews were limited to three states, potentially missing broader national perspectives.
- Virtual interviews in Kerala versus face-to-face interviews in Haryana and Chhattisgarh may have introduced variability in the responses.

## Background

 India is committed to achieving universal health coverage (UHC) by 2030, goal 3.8 of the Sustainable Development Goals.^1^,^3^ As one of the landmark initiatives to help achieve UHC, and part of the government’s flagship health programme—Ayushman Bharat, India launched a nationwide tax-funded health insurance scheme—Ayushman Bharat Pradhan Mantri Jan Arogya Yojana (AB PM-JAY), in 2018.^4^ More than 26 000 public and private providers have been empanelled with the scheme to deliver quality healthcare. Providers are reimbursed a fixed rate under a case-based payment scheme with bundled services defined in individual health benefits packages (HBP).^5 6^ The National Health Authority (NHA) has been endowed with the responsibility for the overall implementation of the scheme including regular updating of the list of HBPs and setting appropriate provider reimbursement rates for these packages.

At the time of the inception of PM-JAY, the HBP reimbursement rates were set through a process that reviewed reimbursement rates in existing publicly financed insurance schemes and the limited available cost data, as well as multiple consultations with stakeholders and experts.^7^ For efficient planning and delivery of services, it is recommended HBP reimbursement rates are set at levels that cover the costs of provisioning as well as incentivise providers to perform in line with PM-JAY’s goals. As cost data has become increasingly available, the rate-setting processes under the scheme have evolved. A series of refinements have been undertaken that include the consideration of nationally representative estimates of healthcare delivery costs collected in a national costing exercise.^8 9^ The estimation of these healthcare delivery costs employed a mix of top–down and bottom–up micro-costing approaches. The top–down method uses retrospective healthcare expenditure data, dividing total costs by the services provided within a specific period, offering a broad expense overview. The bottom–up approach disaggregates data to detail each resource used, providing higher accuracy but is more time-consuming.^10 11^

One of the challenges of the scheme is that, the current reimbursement rates offer a flat rate for each HBP without any regard to the severity and complexity of the cases or any option for adjusting the costs based on individual patients.^12^ To address this, PM-JAY envisions a transition from the uniform case-based payment system to a more refined provider payment system which is also sensitive to patient characteristics.^9 13^ Such a refined payment schedule which reimburses providers for treating patients based on their clinical severity, complications and comorbidity will require robust patient-level data on healthcare costs incurred on their treatment to ensure credible and acceptable reimbursement rates.

Reimbursement rates also need to be updated regularly, which in turn is contingent on the availability of robust and timely evidence on healthcare costs requiring routine collection of cost data.^14 15^ Countries from around the world have approached this challenge in different ways (see online supplemental annexure 1). Their experiences demonstrate that conducting large-scale costing studies can be a useful starting point but they are extremely time and resource-intensive, and the evidence quickly becomes outdated.^14 15^ Additionally, the limited awareness and capacity regarding the use and benefits of healthcare cost accounting systems in lower and middle-income countries (LMICs) exacerbate the problem in these settings.^16 17^ Building sustainable systems that can provide routine cost estimates with limited additional effort from healthcare providers can help address these challenges.

The NHA launched a pilot in April 2022 to establish a sustainable hospital-based patient-level cost surveillance system for generating data related to healthcare resource utilisation and provide insights for guiding reforms in provider payment mechanisms and the rationalisation of the HBP. As part of the pilot, two sets of data are being developed. First, patient characteristics such as age, morbidity, comorbidity, complications and intensive care use are being collected, with morbidity being classified using the International Classification of Disease (ICD)-11.^18^ Second, patient-level data on the quantity and price of resources consumed are also being collected. This includes data on variable inputs of drugs, consumables, implants and diagnostics as well as information on length of stay to account for the cost of fixed resources. Together, the resource use data will be used to determine price weights for patients with the same disease condition but varying severity. The data is entered by the hospitals using the PM-JAY transaction management system (TMS), originally designed for preauthorisation and claim submission.

This study aims to identify the challenges and successes in implementing such a cost-surveillance system. We conducted a qualitative process evaluation of the hospital cost reporting to describe processes followed and challenges faced by the providers as well as the payer agency (PM-JAY) and develop recommendations for setting up such a system in India as well as other LMICs. The process evaluation of the cost-surveillance pilot will also inform the feasibility and potential development of a diagnostic-related group (DRG)-type provider payment system in the future.

## Methods

### Study context

**Figure 1:**
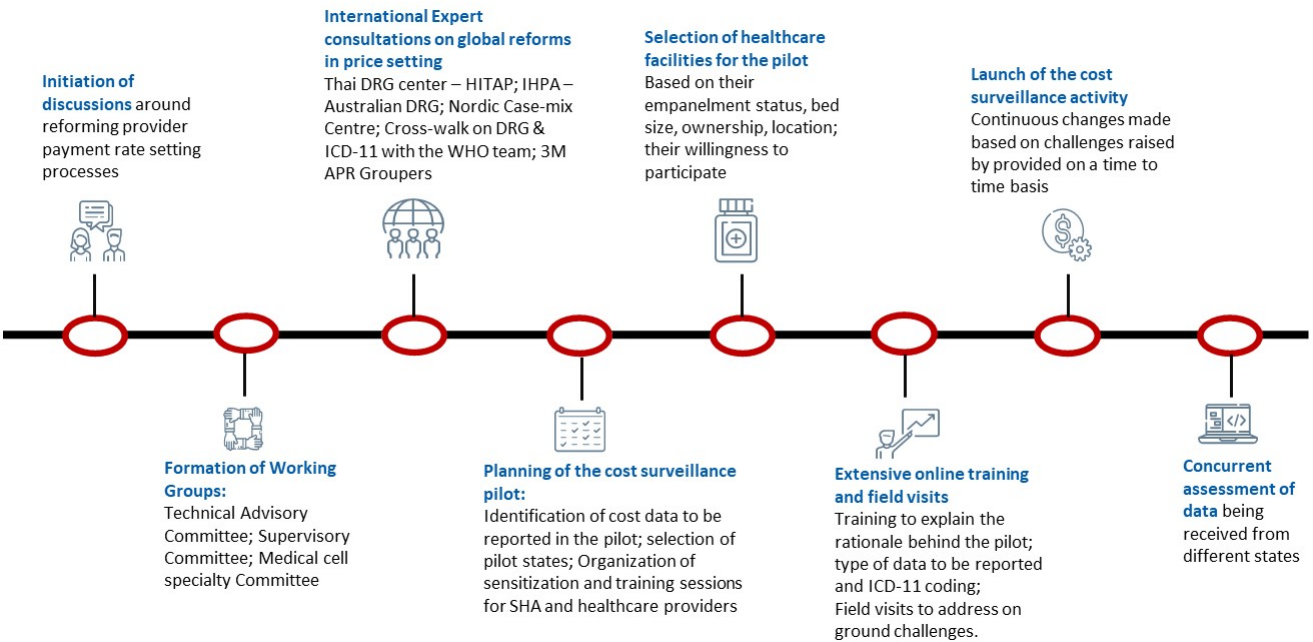
The initiation of India’s healthcare cost surveillance pilot. DRG, diagnostic-related group; HITAP, Health Intervention and Technology Assessment Program; IHPA, Independent Hospital Pricing Authority; ICD-11, International Classification of Disease-11; SHA, state health authorities.

The hospital cost surveillance pilot was initiated in five states (Haryana, Kerala, Chhattisgarh, Maharashtra and Kerala). The NHA underwent an extensive preparatory phase to ensure the smooth commencement of the pilot (figure 1). A Technical Advisory Committee and Supervisory Committee were established and consultations were conducted with experts, country-level payer organisations and developmental partners to comprehensively explore and understand provider payment systems implemented in various countries.

To ensure standardised reporting of morbidity information, the World Health Organisation (WHO) ICD-11 was selected for the systematic recording and classification of diagnosis-related data.^18^ The ICD developer team from WHO trained staff from the NHA and state health authorities (SHA) in the ICD framework. To standardise information on drugs and consumables, Systematized Nomenclature of Medicine- Clinical Terminology (SNOMED CT) system, was used.^19^ For standardisation of diagnostic-related information, the Logical Observation Identifiers Names and Codes database, designed to identify test results and clinical observations accurately, was used.^20^ Each of these systems was integrated into PM-JAY’s TMS to enable hospital-level data entry. Training sessions were conducted among the SHA and the hospital staff in all pilot states, followed by field visits. Subsequently, the hospital cost surveillance pilot was initiated in a phased manner, ensuring a systematic and organised approach.

### Study design and sampling

We employed an open-ended, descriptive and qualitative study design using in-depth interviews (IDI) as the data collection strategy.^21^ This study design was chosen to gain detailed insights and feedback from the providers on the current surveillance system being piloted. We also captured the view of the NHA and SHA representatives responsible for the roll-out and operational aspects of the hospital cost surveillance pilot. Out of the five states where the hospital cost surveillance was initiated, we sampled Haryana, Chhattisgarh and Kerala based on the pilot’s initiation date and their geographical location. We obtained a list of PM-JAY empanelled providers in these states from the NHA, which included information about the providers’ ownership, number of beds, enrolment status for the hospital cost surveillance pilot and their accreditation status by the National Accreditation Board for Hospitals & Healthcare Providers (NABH). We selected seven facilities from each state to ensure a representative sample of public, private and trust hospitals with different bed capacities and NABH accreditation status and a combination of hospitals who had agreed to participate in the pilot as well as those who had not agreed to do so. We interviewed three representatives from the NHA and five officials from the SHA in the three sampled states, those who were responsible for overseeing the activities related to the cost surveillance pilot. Table 1 below presents the characteristics of the facilities chosen for the interviews.

**Table 1:** Characteristics of the health facilities included in the study sample

No.	Health facility Unique ID	Ownership	Enrolment in pilot	Bed strength	NABH accreditation	Specialties	Digital maturity
1	I-PI001	Public	Yes	186	Yes	Dermatology, emergency, dialysis, general medicine and general surgery, obstetrics and gynaecology, ophthalmology, orthopaedics, ear-nose-throat (ENT), paediatric management.	Digital-only billing module.
2	I-PI002	Trust	Yes	30–35	No	General medicine, general surgery, intensive care, infectious diseases, obstetrics and gynaecology, orthopaedics.	Digital-only billing module.
3	I-PI003	Private	Yes	400	No	Burns management, cardiology, dermatology, emergency and trauma, general medicine, general surgery, intensive care, infectious diseases obstetrics and gynaecology, psychiatry, neonatal care, neurosurgery, neurology, nephrology, ophthalmology, oral and maxillofacial surgery, orthopaedics, ENT, paediatric medical management, spine surgery, urology.	Digital-have their own hospital management information system (HMIS).
4	I-PI004	Private	Declined	1000	No	Cardiothoracic surgery, dermatology, emergency, dialysis, general medicine and general surgery, obstetrics and gynaecology, ophthalmology, orthopaedics, ENT, paediatric management, infectious disease, intensive care, psychiatry, neonatal care, neurosurgery, neurology, nephrology, oral and maxillofacial surgery, respiratory and palliative medicine.	Digital-have their own HMIS.
5	I-PI005	Trust	Yes	436	Yes	Cardiology, dermatology, emergency, general medicine and general surgery, infectious diseases, interventional radiology, psychiatry, medical oncology, neonatal care, neurosurgery, neurology, nephrology, oral and maxillofacial surgery, orthopaedics, ENT, organ and tissue transplant, paediatric medical management and paediatric surgery, plastic and reconstructive surgery, respiratory medicine, spine surgery, urology, palliative medicine, ophthalmology.	Digital-have their own HMIS.
6	I-PI006	Private	Yes	300	Yes	Burns and cardiology, dermatology, emergency, general medicine and general surgery, obstetrics and gynaecology, ophthalmology, orthopaedics, ENT, paediatric management, infectious diseases, intensive care, psychiatry, neurology, nephrology, respiratory medicine, surgical oncology, urology, palliative medicine.	Manual reporting (financial records maintained and submitted for audits).
7	I-PI007	Public	Yes	3025	No	Burns, cardiology and cardio-thoracic and vascular, dermatology, emergency and trauma services, general medicine and general surgery, obstetrics and gynaecology, ophthalmology, orthopaedics, ENT, paediatric medical management, paediatric surgery, infectious diseases, intensive care, psychiatry, neurology, nephrology, respiratory medicine, surgical oncology, urology, palliative medicine, interventional neuroradiology, medical oncology, neo natal care, neurosurgery, oral and maxillofacial surgery, organ and tissue transplant, plastic and reconstructive surgery, radiation oncology, spine surgery.	Manual/paper based.
8	II-PI001	Public	Yes	300	No	Cardiology, orthopaedic, neurosurgery, medicine, 24 hours emergency, oncology, gastroenterology, pulmonology, nephrology, obstetrics and gynaecology, paediatric, general surgery, proctology, ENT, outpatient department (OPD), pathology, radiology.	Digital-only billing module.
9	II-PI002	Public	Yes	350	No	Obstetrics and gynaecology, emergency and trauma services.	Digital-only billing module.
10	II-PI003	Private	Declined	120	Yes	Cardiology, orthopaedic, neurosurgery, medicine, 24 hours emergency, oncology, gastroenterology, pulmonology, nephrology, obstetrics and gynaecology, paediatric, general surgery, proctology, spine surgery, ENT.	Digital-only billing module.
11	II-PI004	Public	Declined	2100	No	Forensic medicine, pathology, medicine, surgery, paediatrics, obstetrics and gynaecology, orthopaedics, respiratory medicine, radiation oncology, dermatology, respiratory medicine, burns and plastic, cardio thoracic vascular surgery, cardiology, endocrinology, gastro enterology, neurology, endosurgery, neurosurgery, neonatology, urology, pulmonary and critical care medicine, paediatric surgery.	Digital-only billing module.
12	II-PI005	Private	Yes	150	yes	Oncology, cardiology, medicine, minimum access surgery, orthopaedic, anaesthesia, cardiology and cardio-thoracic surgery, critical care, dermatology, emergency trauma, ENT, gastro-enterology, neonatology, nephrology, intensive care unit services, paediatrics, neurosurgery.	Digital-only billing module.
13	II-PI006	Private	Yes	810	yes	ENT, ophthalmology, general medicine and general surgery, obstetrics and gynaecology, orthopaedics, psychiatry, pulmonary medicine, paediatrics, radiology, dermatology, burns, plastic and reconstructive surgery, dentistry, urology.	Digital-have their own HMIS.
14	II-PI007	Private	Yes	1020	No	Forensic medicine, community medicine, ophthalmology, ENT, obstetrics and gynaecology, surgery, anaesthesia, orthopaedics, medicine, dermatology, psychiatry, chest and tuberculosis (TB), radiation oncology, medical gastroenterology, urology, neurosurgery and radiodiagnosis.	Digital-only billing module.
15	III-PI001	Private	Yes	415	Yes	Dental Surgery, cardio thoracic surgery, orthopaedics, neonatology, paediatrics, general medicine, criticalcare, paediatric cancer, burns, plastic and reconstructive surgery, neuro surgery, cardiology, nephrology, neurology, chest diseases and respiratory medicine (pulmonology), polytrauma, paediatric surgery, surgical oncology, medical oncology, genitourinary surgery, general surgery, ENT, obstetrics and gynaecology.	Digital-only billing module.
16	III-PI002	Public	Declined	1200	No	Chest diseases and respiratory medicine (pulmonology), neurology, cardiology, neonatology, general medicine, critical care, paediatric cancer, polytrauma, burns, plastic and reconstructive surgery, medical oncology, surgical oncology, euro surgery, genitourinary surgery, paediatric surgery, cardio thoracic surgery, orthopaedics, obstetrics and gynaecology, ophthalmology, general surgery.	Digital-only billing module.
17	III-PI003	NGO	Yes	100	No	Paediatric surgery, surgical oncology, burns, plastic and reconstructive surgery, critical care, general surgery, paediatrics, neuro surgery, neurology, general medicine, orthopaedics, cardiology, medical oncology, chest diseases and respiratory medicine (pulmonology), nephrology, neonatology, obstetrics and gynaecology, genitourinary surgery, ophthalmology, cardio thoracic surgery, paediatric cancer.	Digital-only billing module.
18	III-PI004	Private	Yes	30	No	Paediatrics, paediatric surgery.	Digital-only billing module.
19	III-PI005	Public	Yes	500	No	Obstetrics and gynaecology, general medicine, general surgery, dental surgery, ENT, ophthalmology, orthopaedics, polytrauma, burns, plastic and reconstructive surgery, paediatrics, neonatology, chest diseases and respiratory medicine (pulmonology), nephrology.	Digital-billing module and prescription.
20	III-PI006	NGO	Yes	100	No	Cardio thoracic surgery.	Manual/paper based.
21	III-PI007	Private	Declined	50	No	General medicine, chest diseases and respiratory medicine (pulmonology), obstetrics and gynaecology, cardio thoracic surgery, paediatric surgery, critical care, neonatology, cardiology, neurology, nephrology, general surgery.	Digital-have their own HMIS.

Declined: The provider hospitals which were invited to participate in the cost surveillance pilot but declined to do so.

NABHNational Accreditation Board for Hospitals and Healthcare Providers

To strengthen the evidence, a virtual panel discussion was organised involving 32 experts from various sectors, including government agencies, non-governmental organisations, the private sector, healthcare industry partners and the health insurance industry. These experts were invited to review and comment on the initial findings from the IDIs and offer additional insights and viewpoints on the main research questions.

### Tool development and overview of the themes

Three open-ended tool guides were developed, one each for the NHA and SHA officials and the third one for the healthcare providers. The tool guides (online supplemental annexure 2) were developed and piloted beforehand to ensure that all key areas were covered. The tool was developed collaboratively by SS, YC and DS. To ensure its validity and reliability in gathering the necessary information, it was reviewed and revised multiple times by SP and LG. A pilot was conducted by administering the tool to three health facilities: a private tertiary hospital, a government tertiary hospital and a government district hospital. Feedback was incorporated at each stage to refine the questions for clarity and ease of understanding. The pilot phase concluded when no significant feedback or difficulties in comprehension were reported by the providers.

For the NHA and SHA officials, the key themes included in the tool guide focused on the roles and responsibilities of the NHA and SHA for implementing the pilot, the criteria for state and hospital selection and challenges faced while enrolling the hospitals as well as implementing the pilot. The key themes for the healthcare providers were aimed at understanding the overall process of cost surveillance data entry, the sources of data for cost surveillance, challenges faced in reporting for the pilot and the recommendations to improve the overall process for better sustainability. In addition, a structured survey was conducted among the healthcare providers which aimed to capture the difficulty level in cost reporting activity according to the type of information being reported, that is, for quantity and price of resources consumed including drugs, consumables, implants and diagnostics.

### Data collection and analysis

The interviews were conducted in virtual and face-to-face modes, depending on the convenience of the participants. The IDIs for NHA officials and all providers in Kerala were conducted virtually whereas all the interviews in Haryana and Chhattisgarh were conducted face-to-face by making visits to different facilities. Consent to participate and to record the interviews was sought before the initiation of the interviews. All the interviews were audio and video recorded followed by verbatim transcription. Hand-written notes were generated wherever the provider hospitals (12/21, 57%) did not provide consent for recording. All the interview audios, videos and transcripts were given codes to maintain anonymity, the access of which was limited to the authors. The accuracy of the transcripts was verified by the interviewer, who also transcribed any portions of the interviews that were difficult to understand. The details of the individuals were deleted and a unique code was given to the transcript before sharing it with all the authors.

**Figure 2:**
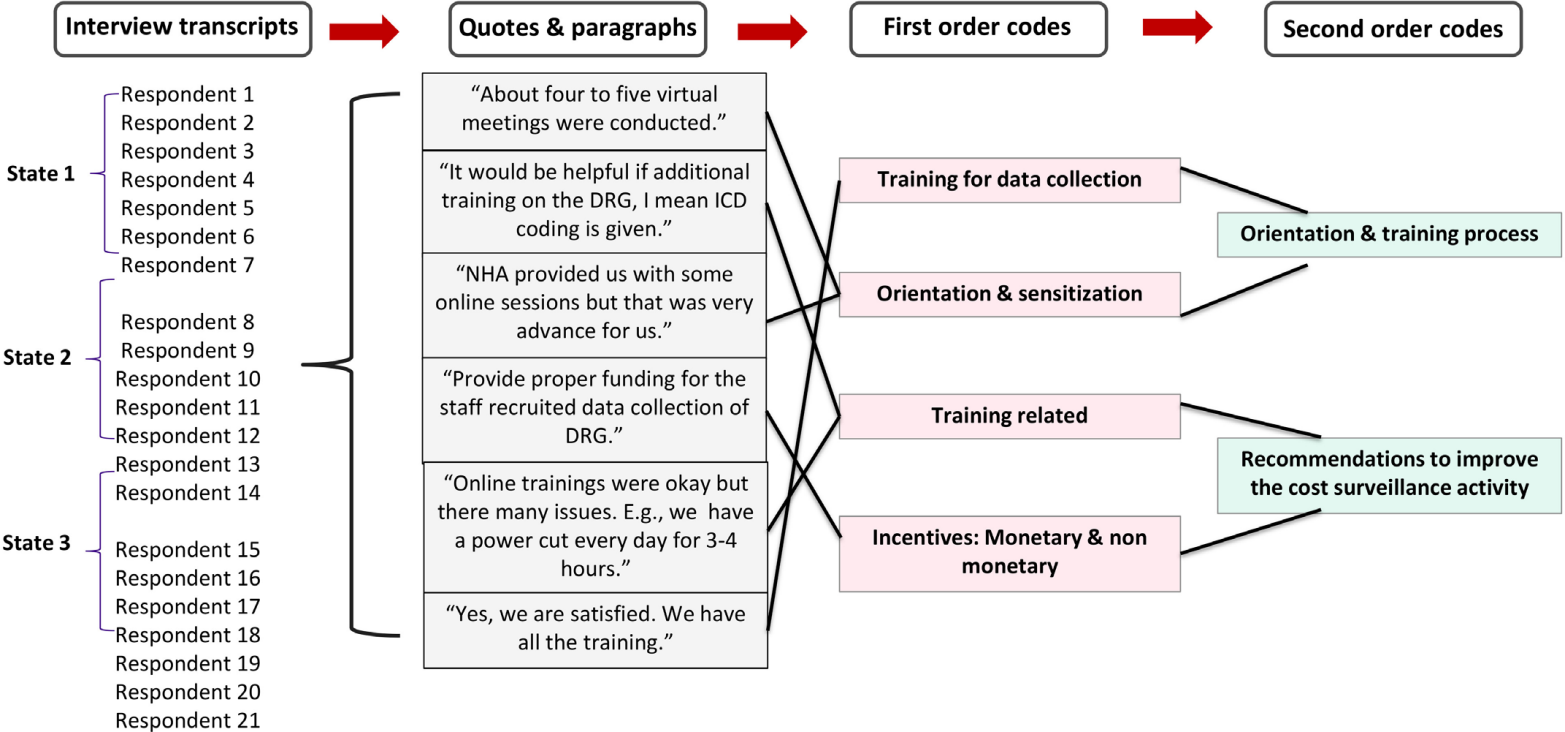
Coding framework for analysis. DRG, diagnostic-related group; ICD, International Classification of Disease; NHA, National Health Authority.

The analysis followed a thematic approach across the interviews to make the comparisons.^22^ A coding framework (figure 2) was developed to add, delete and merge various quotes to identify a priori themes from the tool guide as well as the emergent themes that were derived from the perspective of research participants. Microsoft Excel V.2019 was used to categorise and code the data gathered from all the interviews. Coding was done by SS and YC and in case of any disagreement, it was resolved by LG and SP. All the transcripts were checked for accuracy by LG, DS and SP. Each theme was colour-coded, which was used to select the related transcript excerpts, and was finally tabulated for ease of analysis. The authors reviewed and re-read the excerpts multiple times to advance the interpretation process beyond any single person’s viewpoint. To better understand the challenges, findings are discussed, using an implementation science framework considering acceptability, fidelity and feasibility—factors that ultimately guide the sustainability and scalability of an intervention or programme.^23^

The interviews were conducted by two of the authors: YC and SS and both of them have experience in conducting qualitative research including IDI and focus group discussions in Indian healthcare settings. Both YC and SS hold master’s degrees in public health with experience of 5 and 4 years, respectively. Both of them have been working around generating evidence and support for the rationalisation of PM-JAY health benefit packages.

### Patient and public involvement

Patients or the public were not involved in the design, or conduct, or reporting, or dissemination plans of our research. The interviews were conducted among provider hospital staff which included medical superintendents, medical directors, data entry operators, administrative in-charge, hospital owners, as well as the accounts and finance officers.

## Results

### Participants characteristics

**Figure 3:**
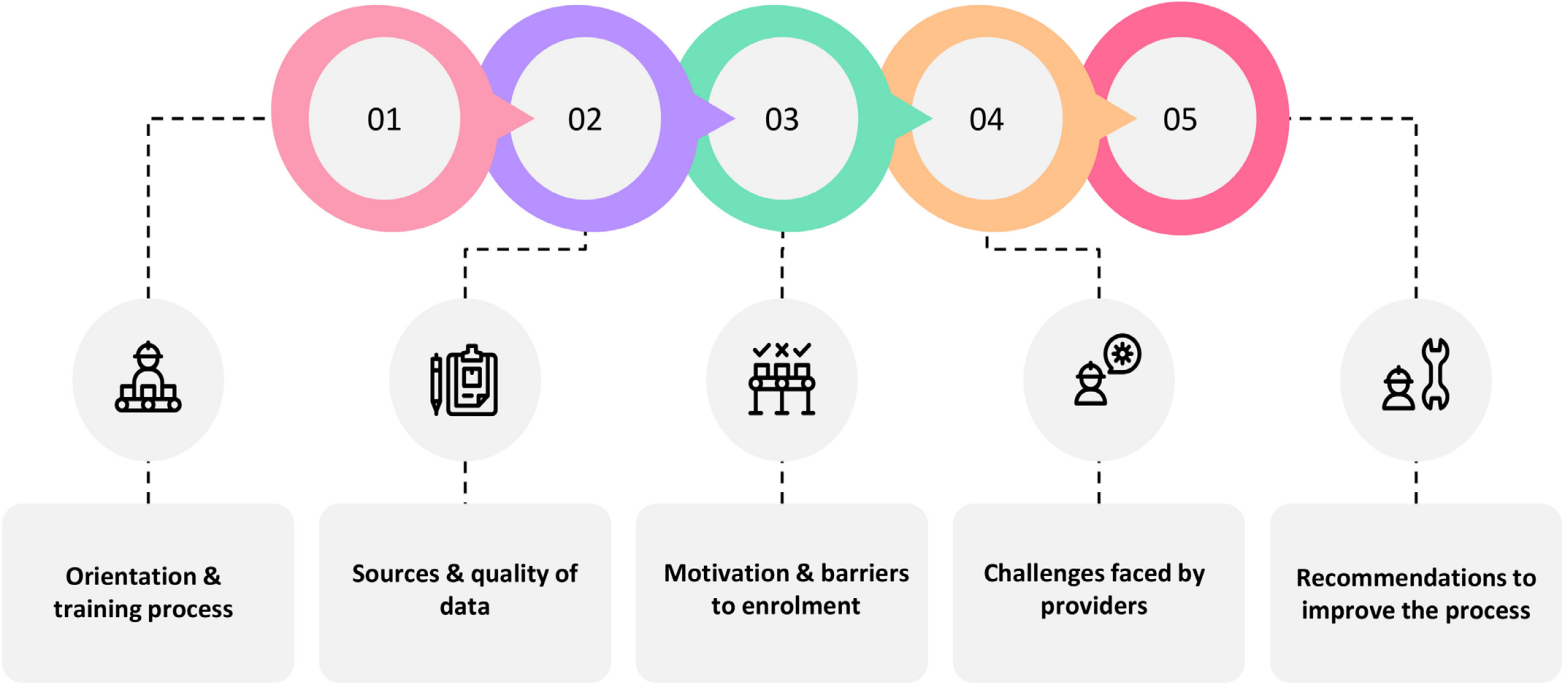
A priori themes identified from the interview tool guide.

Within each health facility, the interview respondents included the medical superintendent and medical directors (15%), data entry operators and Pradhan Mantri Ayushman Mitra (PMAM), that is, the official responsible for facilitating patient registration to access treatment and enter data in the TMS (46%), administrative in-charge (7%), hospitals owners (17%) as well as the accounts and finance officers (15%); table 2. Among the NHA and SHA representatives, those responsible for the roll-out and operational aspects of the hospital cost surveillance pilot were interviewed. The subsequent section provides detailed thematic analysis findings (figure 3).

**Table 2:** Roles and responsibilities of the respondent interviewed across the study sites

No.	Respondent roles/Responsibilities	Site I	Site II	Site III	Total
1	Medical superintendent/medical director	3	2	1	6 (15%)
2	Data entry operator/PMAM*	5	7	7	19 (46%)
3	Hospital owner	2	3	2	7 (17%)
4	Administrative staff	1	1	1	3 (7%)
5	Finance and accounts personnel	1	2	3	6 (15%)

* PMAM: Pradhan Mantri Ayushman Mitra.

### Key findings

#### Training and sensitisation sessions for the cost surveillance pilot

The rollout of the hospital cost surveillance pilot was preceded by orientation and training sessions organised by the NHA for the SHAs as well as the providers. The orientation sessions for the SHAs were held to help them develop an understanding of the need to reform provider payment rates and the concept of DRG-based payments. They were also sensitised regarding the data that needed to be collated and entered into the TMS by the healthcare providers. Further, a team of NHA and SHA officials was identified to train the hospital staff for the cost surveillance activity. All the providers as well as SHA officials echoed that, ‘multiple layers of sensitization session were organized [by the trainers] in form of presentations, online meetings and [followed by] discussions’; NHA.

The interviews revealed mixed views on the effectiveness of the implementation of the orientation and training sessions for healthcare providers. A number of virtual trainings were conducted that catered to the staff responsible for the cost surveillance data entry at the providers. The SHA officials agreed that multiple rounds of training were conducted for both SHA staff and healthcare providers, which included data entry training and ICD-11 coding training for the SHA staff: ‘When [the pilot was] launched, then Dr xxxx did the sensitization of all the hospitals again. Three rounds of training sessions were provided by NHA, [and] three rounds were provided for ICD training’, SHA, Site III. However, even though the majority (20 out of 21) of the health facilities confirmed that at least one training session had been conducted, one of them reported that they do not remember such sessions; ‘I don’t remember exactly maybe we would have got a session as well but we received some documents on to which they wanted our consent’; data entry operator/PMAM; Site II.

Second, the SHA officials as well as the providers felt that the online sessions organised were brief and not sufficient to enhance the skills of the staff but would require rigorous physical training: ‘in the SHA at the state level [the staff] was not able to understand that [training content] very well. And as a result, when we [the SHA staff] were training the hospitals online or through Zoom only, [so] again, there was this gap in communication’; medical officer; Site III. Given this, the majority (17/21) of providers mentioned that virtual training was not sufficient ‘No [physical] training [was conducted], I will be very honest, that [virtual training] is not sufficient’; data entry operator/PMAM; Site I.

Third, another issue relates to the time gap between the training and the actual start of this surveillance exercise. ‘It was okay, they trained us twice. But the time between the training and the actual launch was a few months, so that might be the case’; medical officer; Site III. Further, respondents from more than half of the hospitals (16 out of 21) interviewed highlighted that the online training sessions were mostly attended by the higher staff and not the data entry operators ‘See, we were also the part of same sensitization session, it was one and half hour of session and we attended the same session so may be the owner joined the training, nodal officer attended it and the person who is supposed to do it was not part of it. This might be the case. They are so overburdened that they can’t focus on everything’; data entry operator/PMAM; Site II.

#### Sources and quality of data used for cost surveillance

The providers were interviewed to understand the sources of information for the cost surveillance data entry. The consensus among the participants was that a combination of the patient case sheet, information from hospital information systems (HIS) and pharmacy bills were used for the data entry information. While in the public health facilities, patient case sheets were used, the private providers used HIS in addition to case sheets and pharmacy bills ‘So, they [provider hospitals] have a hospital software, basically their own version of the HMIS [hospital management information system], which is available, and they have the cost, they’re getting the cost details from the software system’; accounts/finance officer; Site I.

In terms of mechanisms in place to ensure the quality of data that is being entered into the TMS, the providers mentioned that the data quality is ensured by cross-checking with case sheets ‘first we enter [data] from bills, so we cross check the data and second is from the medication chart’ as well as a dedicated team that has been deployed to ensure data quality ‘We have authorized, one clerk and then PRO [public relations officer] is monitoring this always’; administrative in-charge; Site II. However, a few hospitals have reported that there is no such quality assurance mechanism as the ongoing cost surveillance is in pilot mode as of now ‘No, there is no team. It’s a pilot project that’s why there is no checking’.

#### Motivation and barriers guiding healthcare provider enrolment

Among those empanelled providers who agreed to join the pilot initiative, 3 out of 21 agreed that even if they do not see the immediate benefit, they believe this will benefit in later stages ‘So, for our own organization I don’t see that, it’s not an immediate benefit for us but I think from a public health point of view it’s going to be an informative exercise to know what it costs, what it takes to do something, so it is from that point of view that it is a useful exercise’; medical superintendent; Site III. Similar to this, a trust hospital appreciated the initiative and also agreed that this activity has led them to collect this information and now it has become easier to track their spending ‘By collecting all this data, we can tell anyone regarding how much medicines have been used on the patients. Because we do not charge patients for anything so we had no idea [of] how much we were spending on a single patient*.* This gives us an idea too’; hospital owner; Site III. Further, two of the providers mentioned that they joined out of a sense of social responsibility ‘Also, we being a medical college with all the staff and facilities, if we don’t come forward then who else will’; medical officer, Site I.

While exploring the potential reasons for declining to participate in the pilot, the providers raised two major concerns, one, they had limited clarity on why this initiative was being undertaken ‘Yes, we received one e-mail at that time [but] it was not clear why they were doing this. So, we refused to be a part of this’; hospital owner; Site II. Second, the providers were also constrained by time and shortage of staff ‘The hospital rejected due to time constraints, as we already have so much work and other was lack of knowledge’; ‘We have shortage of manpower. We just can’t give this work to anyone’; administrative in charge; Site II.

#### Challenges

The providers described multiple challenges that they faced in the initiation of the pilot. First, there was a lack of willingness to participate among the providers driven by a lack of understanding ‘I have asked them so many times that you suggested our name for the DRG but tell us why are we doing this, what’s the benefit. No one has any idea’; hospital owner; Site III. Second, the hospitals felt an increase in workload in a context that is already short of human resources ‘Hospitals are refusing to take part in this saying that we don’t have enough manpower. This is just extra work for them’; medical director; Site II and, further, that it duplicates existing work as they already collate this information for their HIS. The providers reported that on average it takes about 2 hours to complete the cost surveillance data entry for inpatient admission, with a length of stay of 3–5 days. This means that in high-volume centres, there would be an average of 30 (19–65) additional person-hours required per day per hospital. To meet the increased workload, six hospitals had to hire additional staff and two hospitals had to purchase new systems, yet they were still struggling to submit the required information on time. One hospital has asked their nurses to enter the data in an Excel sheet whereas others have trained their current staff.

Third, there is a lack of trained human resources to meet the needs of the data entry. The cost surveillance data entry is contingent on understanding clinical information including the entry of primary and secondary diagnoses using ICD-11 coding as well as information related to the drugs and diagnostics. To fill out such information, some degree of clinical understanding is also required ‘PMAM could not understand the primary diagnosis and secondary diagnosis’; medical officer; Site II. However, at all the facilities interviewed, the staff deployed for entering the cost data ‘are 12th pass and can you expect from him that he can check the sub-types of malaria’; administrative in charge; Site I. With skills limited to data entry processes so that ‘Even if 6 months training is provided, then also we cannot read because it’s not related to our field’; data entry operator/PMAM; Site II. The lack of clinical understanding leads to incomplete information and compromises the quality of the data entered. The staffing problem is exacerbated by a high turnover of data reporting staff in these hospitals: ‘Again, the biggest problem is PMAM [data entry staff] keeps on changing. We have given so many trainings and we know how many PMAM have left. So new PMAMs have joined and they have not received the trainings’; SHA; Site III.

It was also highlighted that the trainings were attended by the higher staff and not the data entry operators who were responsible for data entry. More importantly, the SHA officials also revealed that there was a gap between the training session and the initiation of the pilot which led to some dissipation in knowledge. In addition, the capacity building sessions were theoretical as the TMS fields and its integration with ICD happened at later stages ‘So, capacity building training sessions were done, then NHA provided ICD 11 training sessions in March 2022 to SHA. Then ICD was integrated in the system around June or July’; ‘In the month of February- March 2022, training sessions of ICD were conducted. For DRG it was in April. But It (DRG) was started in the end of October or in the first week of November 2022’; SHA; Site II.

Fourth, ‘The overall speed of the TMS portal is slow actually’; data entry operator/PMAM; Site III. The majority of participants identified various issues with the TMS (transaction management system) used for data entry and reported difficulties ranging from the TMS being too slow to not working at all in rural areas. One-third of the participants reported having to re-enter data because the page or session would expire in a few instances ‘If long time is taken then the session expires and we have to reload the page’; data entry operator/PMAM; Site I. 15 out of the 21 facilities reported that it is difficult to enter the medicine and diagnostics-related information as the TMS offers only the generic names and the case sheets mention brand names ‘Syrup components do not match, brand name option is not there, we have just components name there, [we cannot find] some medicine names’; data entry operator/PMAM; Site II.

**Figure 4:**
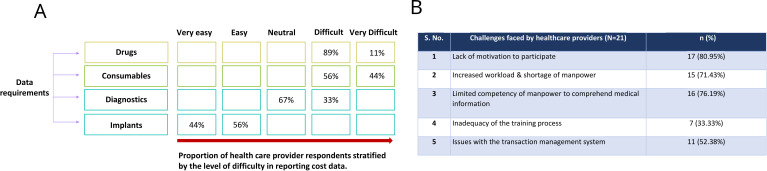
Difficulty in reporting healthcare cost data to the transaction management system.

The providers reported that capturing the details on drugs and consumables for the entire stay of a patient was either difficult or very difficult while filling out the details on diagnostics and implants was relatively easier (figure 4A). The challenges have been summarised by the number of providers facing each of them as figure 4B.

#### Recommendations

First, all the providers echoed that physical training sessions rather than virtual ones should be conducted to improve the training process for data entry staff. One of the SHA officials also suggested that, given the complexity and ability of the data entry concerning medical information, more workshops specific to ICD coding are required ‘I think course work is needed. We need people who are trained specially in this and they [data entry staff] get certified and do this work’; SHA; Site II. Second, eight participants suggested that the data submission process should be revised by either allowing to upload scanned documents so that data is entered centrally or allowing for an offline standardised MS Excel-based data entry form which could then be submitted ‘There should be a system for bulk upload in excel. We have a system of data entry in a single excel sheet regarding utilization of medicines, so it is unnecessary duplication of the work’; ‘there would be a separate excel sheet where we [data entry staff] would need to enter the details and later this excel sheet could [be] appended with the patient record’; data-entry operator; Site III. Another method which was further suggested by facilities was an automatic submission of data through their HIS.

Third, all three SHA officials stated that it was extremely difficult to motivate facilities to join the cost surveillance pilot without any incentive. All of the facilities agreed that certain additional incentives should be provided. A public hospital urged that the entire team supporting the patients, including administrative personnel, PMAMs and others, be recognised for the amount and scope of work they accomplish. Further, 11 out of the 21 providers requested that if not monetary, other non-monetary benefits like priority accorded to settlement and disbursement of claims and appreciation of the staff involved could be provided ‘They [empanelled hospitals/providers] were asking that if their claims will be approved at a priority or they will get TMS approval at priority’; SHA; Site II.

Finally, given the lack of human resources and the considerable patient volumes, six providers also requested that ‘ [the government] should either provide funds to us [hospital] so that we can hire manpower or send a team on their payroll to collect all the data’; hospital owner; Site II; and that ‘…two human resources’ salary should be there; one for the data entry part and the other for the time of a senior person who can do an audit or quality check to see whatever is entered is correct’; administrative in charge; Site III. They believed that such an incentive would improve the overall quality of the data being collected as well.

All the quotes extracted from the transcripts have been appended as online supplemental annexure 3.

## Discussion

Our paper presents the findings of the initial evaluation of a government initiative led by the NHA in India, which aims to gather data on patient-level healthcare costs at the facility level. Our study findings indicate that reporting cost data at the hospital level poses significant challenges. First, there was a lack of sufficient capacity in existing hospital staff to provide complete and high-quality data. This stems from basic non-medical qualifications, limited direct training to the grassroot functionaries and their frequent turnover. Second, the process is resource and time-intensive, straining already constrained capacity. Third, there are operational issues with the TMS, as data entry operators have expressed concerns about its speed, user-friendliness and frequent page expirations. Finally, the existing patient records do not provide reliable information on the quantity of consumables used and prices of certain inputs.

In terms of acceptability, preliminary findings indicate that the current approach places a significant burden on healthcare providers, leading to hesitance and unease in participation. This can potentially compromise the quality of collected data and affect decision-making in the healthcare sector. The success of provider payment reforms relies heavily on high-quality data.^24^ Several countries have shown that a small but representative sample can be sufficient in the initial stages to generate initial cost weights and refine case-based payments (online supplemental annexure 1).^25^,^28^ In the absence of comprehensive cost data, countries like Thailand, Estonia and Croatia have used charges or hospital reimbursement data as an initial proxy for costs, but charges do not necessarily reflect costs and can be used by providers to skew the payment incentives (online supplemental annexure 1).^25^,^31^ Therefore, it is recommended to start data collection with a limited scope of services and facilities, gradually expanding in phases. This approach acknowledges the challenges posed by extensive data collection requirements and allows for a more manageable and practical implementation of cost surveillance systems. It provides an opportunity to refine the data collection process over time while still obtaining valuable insights for provider payment reforms and decision-making in the healthcare sector.

While assessing fidelity, which implies evaluating adherence to programme requirements, we identified critical factors ensuring data accuracy, despite not having access to the pilot’s collected data. The design of a case-based payment system hinges on the quality of patient records, which depends on accurately distinguishing between different diagnoses and procedures and patient complexity within a single group.^32^ The adequacy of clinical documentation, availability of skilled staff and consistent training influence the accuracy of this information. Based on our findings, it is challenging for data entry operators, considering their competency and qualifications, to accurately classify diagnoses. Therefore, strategies should be implemented to create or enhance the coding workforce.^33^ Several countries like the USA, Croatia, Australia and others developed training and evaluation strategies for coding during their pilot phases (online supplemental annexure 1).^25^,^31^ Additionally, improvements are necessary in clinicians’ prescription practices to ensure accurate mention of diagnosis-related information.^34 35^ Addressing these challenges is crucial to improving the accuracy and reliability of patient records.

Another significant finding pertains to the accurate reporting of information related to drugs and consumables.^14 15^ To address the issue of branded names in medical records versus generic names in the SNOMED-CT terminology system, it is necessary to focus on clinicians and encourage them to improve their prescription practices.^36 37^ Emphasising the importance of prescribing and using generic drug names will contribute to improved transparency in prescription practices, enable smoother data entry processes and align with the requirements of the cost surveillance system.^38 39^

Third, the findings highlight several caveats in the training process. Bridging the gap between training and implementation is crucial. Tailored training sessions, supplemented with supportive supervision, will better enhance proficiency. More importantly, regular monitoring, feedback and guidance to address challenges are pivotal for continuous improvement.

The third key aspect guiding the sustainability and scalability of a programme is its feasibility. Our study reveals that the cost surveillance data entry process, particularly concerning consumables, is notably time-consuming. National costing studies in India have shown that consumables, along with human resources and drugs, constitute a significant proportion of the total cost.^14^ However, there is a lack of disaggregated data on the quantity of consumables, and information on the prices of these items is not available for all of them.^15^ To address this issue, it is necessary to integrate and collate information on consumables in facility health records. This integration would enable a comprehensive understanding of the consumption patterns and costs associated with these items. Efforts should be made to establish systems that allow for efficient recording of consumable data, ensuring that it is easily accessible and retrievable for analysis.^40 41^ Several countries like Thailand, Australia and Germany have developed national costing systems to address such data needs to supplement the provider payment reforms (online supplemental annexure 1).^26^,^30^

In addition to the challenges of disaggregated data and increased workload, the operational efficiency of the TMS significantly impacts the time and effort required for cost surveillance. Data entry operators have raised concerns regarding the cumbersome data entry process, frequent page expirations and the overall speed of the TMS. To address these concerns, one possible solution is to allow offline entry of cost surveillance data in a format acceptable to providers, which can later be uploaded to the system. By enabling offline data entry, healthcare providers can enter the required information at their convenience without the constraints of unreliable internet connections or limited bandwidth.^42^ This approach allows for a more efficient and flexible data entry process, reducing the burden on data entry operators and potentially improving the accuracy and timeliness of the cost surveillance data.^42^ However, it is important to ensure that the offline data entry format aligns with the requirement of the authority to maintain compatibility and facilitate seamless data integration.

### The way forward

Our findings raise doubts about the sustainability of the cost surveillance pilot and the quality of information generated. A potential way to improve the system is to motivate the providers by incentivising them to provide quality information. Non-financial incentives can be used that do not entail additional financial liability for the payer, for example, prioritising claims processing for good performers. To establish a viable and scalable healthcare cost surveillance system, integration with existing billing and patient information systems, as well as management information systems (MIS) is crucial, in particular where providers have already digitised data on drug quantities, prices, consumables, implants and diagnostic tests. Integration would allow for automatic data retrieval through application programming interfaces. However, standardisation and interoperability of existing systems are necessary for successful integration to ensure seamless communication and compatibility.

The ongoing digital transformation in India, driven by the implementation of the Ayushman Bharat Digital Mission (ABDM), presents a significant opportunity to establish a sustainable healthcare cost surveillance system.^43^ Key components of the ABDM, such as the Ayushman Bharat Health Account (ABHA) for unique individual identification, registration of health facilities and professionals and a unified health interface (UHI), can support the creation of a cost surveillance system. The UHI enables interoperability and information exchange between ABDM-compliant health information and patient electronic health records. MIS platforms within the ABDM ecosystem capture comprehensive details including patient issues, symptoms, diagnosis coded as per ICD-11, drug information, diagnostics, implants and procedure-related consumables. More importantly, the ABDM ecosystem has been integrated with the National Health Claims Exchange (NHCX) to facilitate the seamless exchange of data, documents and images between payers including insurance companies, third-party administrators (TPA) or the government scheme administrator and the providers including hospitals, laboratory, polyclinic, others. In addition to the integration with ABHA, health facilities and professional registries, the NHCX is also integrated with the TPA and payer registries. This system verifies the identity of individuals intending to share information, obtains their consent via the consent manager and securely manages the exchange of health records. By leveraging the capabilities of existing systems and the infrastructure provided by the ABDM, a robust and effective healthcare cost surveillance system can thus be established in India. Presently, there are 37 hospital management information system (HMIS) that are compliant with the ABDM. Out of these, six HMIS platforms are government-owned.^43^ The government of India specifically recommends two systems called e-Sushrut and e-Hospital and these systems are primarily being used in government hospitals, but efforts are underway to expand their coverage in the private sector as well.

## Conclusion

Generating cost information for price-setting is a complex and resource-intensive process, however, it is imperative to provide accurate cost data for evidence-informed health policy decisions. Moreover, such critical information is pivotal to encouraging healthcare providers towards more efficient service delivery. The ongoing cost surveillance pilot is a big leap in the direction towards developing robust and evidence-based price-setting processes for the world’s largest insurance scheme. However, to ensure the sustainability of such systems, there is a need to focus on building capacity at all levels of healthcare delivery to accurately capture and report cost data. Further, such systems need to be built into the existing digital infrastructure without posing an additional burden on the healthcare providers, which may otherwise lead to poor quality or incomplete data being captured. The learnings from India’s journey given the challenges being faced and the consistent efforts and strategies being devised by the national authorities to address these concerns provide valuable lessons for other LMICs who are striving to achieve UHC.

## supplementary material

10.1136/bmjopen-2023-082965 online supplemental file 1

10.1136/bmjopen-2023-082965 online supplemental file 2

10.1136/bmjopen-2023-082965 online supplemental file 3

## Data Availability

All data relevant to the study are included in the article or uploaded as supplementary information.
