# Supplementary material for: Establishing national hospital costing systems: insights from the qualitative assessment of cost surveillance pilot in Indian hospitals
Source: BMJ Open. 2024 Sep 10;14(9):e082965. doi: 10.1136/bmjopen-2023-082965 (PMC11409349; doi:10.1136/bmjopen-2023-082965)
Supplement: online supplemental file 1 [file bmjopen-14-9-s001.pdf]

Annexure 1

The evolution of cost data systems informing provider payments in different countries.

**Countries**	**United States (1,2)**	**Australia (1-3)**	**Thailand (1-4)**	**Germany (1-2,5,6)**	**Estonia (1,4-7)**	**Croatia (1,4)**	**China (Beijing) (1,9)**	**Russian federation (1)**
**Initiation of pilot phase for transition to DRG based payment reforms**	In one state (New Jersey) in 1980	1981	1993	2000	2001	2002	2006	2011
**Duration of transition period to DRGs**	10 years	12 years	10 years	3 years	2 years	7 years	5 years	2 years
**Sources of cost data**	Costing studies.<br>In case of unavailable data, length of stay used as proxies for the cost of selected services	National hospital cost data collection system was developed	Used existing hospital charges or health insurance reimbursements	Cost accounting systems were developed	Used existing hospital charges or health insurance reimbursements.<br>Borrowed cost-weights from HCFA-DRG where no. of a particular type of case were low.	Used existing hospital charges or health insurance reimbursements. (Cost data available before the introduction of DRG as patient level electronic invoicing was introduced in 1999)	Used existing hospital charges or health insurance reimbursements (3 years historical charge data)	Costing studies (step-down accounting)
**Assessment during pilot activity**	Annual assessment for several years and were made publicly available and studied widely.<br>Volume of admissions were reported weekly.	-	-	Annual assessments made	Involved relevant stakeholders and experts to regularly review the information collected by making it publicly available.	-	Impact evaluation carried out after one year of the pilot	-
**Training and auditing for data collection and diagnosis related coding**	Training and accreditation of coders, periodic reviews were conducted.	Clinical coding audits in each state hospital system conducted by external organizations	Every time a new DRG version is launched, each insurance scheme organizes training for hospital staff. The training facilitates compliance with the adoption or deletion of these primary classification codes for higher performance of the new DRG grouper software.	-	-	Piloting was supported by international consultants with the main focus being on training of trainers. Training in diagnosis and procedure coding, coding standards, and DRG grouper logic and design principles was carried out, and data was regularly monitored.	Staff responsible for coding and medical personnel were trained on how to code the diagnoses and procedures under the new coding system as well as on how to record discharge data in accordance with the technical guidelines. A monitoring group of experts was established to regularly assess the data quality.	Quarterly monitoring and evaluation workshops in each of the pilot regions; Additional training on step-down cost-accounting methodology; No special training for using the Nomenclature, however, the quarterly monitoring and evaluation workshops discussed the coding related problems.

**Hospitals enrolled for routine cost surveillance**	All Medicare providers	All public hospitals participate every 1 to 2 years. A separate system of data collection is undertaken from 91 (out of 630) private hospitals on a voluntary basis.	Some 900 public hospitals provide data on a routine basis; e-Cost project: patient level HMIS	About 225 volunteering hospitals meeting National cost accounting standards (~13% of all hospitals)	Hospitals contracted with the national health insurance fund (~65% of total health expenditure)	-	Cost data collected from 1400 hospitals	cost-accounting study covering 30 hospitals from three Russian regions was conducted in 2012. In 2014, the cost-accounting study was replicated in 12 regions.
**Frequency of cost data collection**	Annually	Every 1-2 years	Periodical	Annually	Annually	Not refined since 2006	-	-
**Incentives**	-	-	-	Participating hospitals receive a fixed allowance for sharing the cost accounting data.	-	-	-	-
**Additional data entry or data fetching from digital systems**	Data fetching	Additional data entry	Data fetching	Additional data entry	Data fetching<br>Development of an electronic database was done and most local insurance funds had electronic databases using various data delivery systems	Data fetching	Data fetching	Additional data entry
**Type of data being collected for DRG classification**	Patient's principal and secondary diagnosis and other factors affecting the patient's care/ treatment including complications and co-morbidities.	Principal diagnosis, procedure details, Age, Gender, discharge status, birthweight, mental health status, length of stay, comorbidity, complication,	Principal diagnosis, procedure details, Age, Gender, discharge status, birthweight, comorbidity, complication,	Clinical case data (diagnoses, operations, and procedures, gender, age, etc.), additional service data (further service/activity information, e.g., operating room minutes, methods of allocation used, etc.)	Information on all procedures and treatments, kind and amount of the invoiced charges, primary and secondary diagnosis, sociodemographic characteristics of the hospital cases such as age, sex and living region.	Electronic medical records captured in their electronic invoicing systems, adapted to Australian DRG	Medical record information mainly including patient information, diagnosis and treatment data, hospitalization information, expense data, etc. including admission diagnosis, surgical operation, main diagnosis and other diagnoses.	Details on procedures, and patients' discharge information (age and sex of the patient, secondary diagnosis, and length of stay at the hospital)
**Whether cost data collected from same hospitals every time or they are being changed**	All the participating providers	All public and voluntary private providers	All providers under the Universal Coverage scheme	Provider hospitals being added regularly	All providers	-	-	-

**Integration with ICD-10 or any other**	ICD-10-CM	ICD-10-AM	ICD-10	ICD-10-GM	ICD-10	ICD-10-AM	ICD-10 & ICD-9-CM-3	Russian Classification of Medical Services
**Integration with SNOMED CT**	Yes	Yes	-	-	NOMESCO Classification of Surgical Procedures (NCSP)	-	-	-

1. Bredenkamp C, Bales S, Kahur K, editors. Transition to Diagnosis-Related Group (DRG) payments for health: lessons from case studies. World Bank Publications; 2019 Dec 19.
2. Barber SL, Lorenzoni L, Ong P. Institutions for health care price setting and regulation: A comparative review of eight settings. The International Journal of Health Planning and Management. 2020 Mar;35(2):639-48.
3. World Health Organization. Case-based Payment Systems for Hospital Funding in Asia An Investigation of Current Status and Future Directions: An Investigation of Current Status and Future Directions.
4. Mathauer I, Wittenbecher F, World Health Organization. DRG-based payments systems in low-and middle-income countries: Implementation experiences and challenges. World Health Organization; 2012.
5. Tan SS, Geissler A, Serdén L, Heurgren M, Van Ineveld BM, Redekop WK, Hakkaart-van Roijen L. DRG systems in Europe: variations in cost accounting systems among 12 countries. The European Journal of Public Health. 2014 Dec 1;24(6):1023-8.
6. Vogl M. Assessing DRG cost accounting with respect to resource allocation and tariff calculation: the case of Germany. Health economics review. 2012 Dec;2(1):1-2.
7. Haigekassa E. Overview of Estonian experiences with DRG system.
8. Research Data Centre of the federal statistical office [Internet]. [cited 2023 Jun 13]. Available from: https://www.forschungsdatenzentrum.de/en/health/drg
9. Zhang Q, Li X. Application of DRGs in hospital medical record management and its impact on service quality. International Journal for Quality in Health Care. 2022;34(4):mzac090.

ICD-10-CM: International Classification of Diseases, Tenth Revision, Clinical Modification; ICD-10-AM: International Statistical Classification of Diseases and Related Health Problems, Tenth Revision, Australian Modification; ICD-10-GM: International Statistical Classification Of Diseases And Related Health Problems, 10th revision, German Modification; ICD-9-CM-3: International Classification of Diseases, Ninth Revision, Clinical Modification, Volume 3; SNOMED CT: Systematized Nomenclature of Medicine Clinical Terms; NOMESCO: Nordic Medico-Statistical Committee
