# Supplementary material for: Establishing national hospital costing systems: insights from the qualitative assessment of cost surveillance pilot in Indian hospitals
Source: BMJ Open. 2024 Sep 10;14(9):e082965. doi: 10.1136/bmjopen-2023-082965 (PMC11409349; doi:10.1136/bmjopen-2023-082965)
Supplement: online supplemental file 2 [file bmjopen-14-9-s002.pdf]

Annexure 2: Interview Tool guide

Objectives of the health provider interviews

- To understand cost-accounting practices, the role of cost accounting in hospital management, and the capacity for cost accounting in hospitals (public/private/empanelled) in India.
- To understand provider experiences of reporting for the DRG pilot using the PMJAY transaction management system

Interviewer to provide the consent form and request the participant to sign this and agree to the interview being recorded.

Part I: For DRG pilot providers only:

Introductions

1. Could you please tell us about your overall experience of **<u>reporting the new data fields</u>** to the TMS under the DRG pilot?
   a) How and why did you get involved?
   b) How did the orientation process work?
   c) What preparatory steps have you had to make? (training, sensitisation, hospital preparation/investment/recruitment etc)
   d) What are the source(s) for the data that you submit to the TMS? (How are these documented within your organisation?)
   e) How does the reporting process work now that it is up and running?
   f) How do you assure for the data quality before submitting it? What are the steps involved in this?
   g) Please can you describe the auditing/quality assurance process implemented by the NHA/SHA

2. Does reporting the data to the TMS in this way provide any benefits to your organisation? In what way?

Probe:<br>a. Can you please provide your comments on benefits of collating and reporting data with hospital.

b. Can you please provide your comments on the quality of the data collected and generated by the health facility.

3. Can you tell us about any particular challenges and how did you overcome those?

Hints:

a. Challenges in setting up the systems/ capacity/ HR
b. Challenges in reporting- reporting same data to multiple insurance providers, now DRG?
c. Challenges in quality assurance processes
d. Any challenges or complaints by patients during the time of DRG pilot? (More time spent on giving explanations, quality of care, premature discharge, and frequent readmissions?)
e. Any challenges related to coding? How do you make sure that the coding is correct? Do you have multiple checks or were any support provided while coding?

4. Do **you think the full set/other empanelled providers will find the additions to the TMS acceptable and feasible?**

5. What would be your recommendations for improvement in the transaction management system (TMS) in regard to collecting the new data if this was to be rolled out to all empanelled providers?

Part II: Interview tool

1. How do you feel about the reimbursement rates set by PMJAY? How could they be improved and what would be the best process for doing so?

2. Imagine you were the person to set reimbursement prices for PMJAY, what other information would you use to identify the correct amount? Do you see a role of hospital financial information in setting prices?

3. What is the main purpose of maintaining finance and cost information/records in your facility/organisation as it is currently done (e.g., reporting only; management tool; price/charge setting?) Please tick all that apply.
    - Management tool
    - Financial reporting

- Regulatory reporting
- Price-setting
- Budgeting
- Other (please specify)

4. Do you/have you hired any external consultancy (private) for cost accounting. If so, please describe their role, when this was and what their output was.

5. Please list the types of data and their sources that are collected to inform the cost accounting /billing/financial reporting? Who is the person responsible for providing these data?
6. Do you have any standardized templates for the cost accounting/financial reporting processes? Please can you describe these?
7. Can you elaborate on how do you estimate and segregate costs in your hospital system? How are your financial reports broken down (eg. by line items, by department, by patients or a combination of these).

8. Does this process vary by different departments in your hospital? How does this vary?

9. **(OPTIONAL)** Which kind of costing system(s) do you use?

   - Direct costing systems (only direct costs are assigned to cost centres/objects)
   - Absorption (full costing)
     (a) Allocation based on traditional direct absorption costing
     (b) Allocation based on ABC costing
   - Other … please specify

10. How do you record the data required for your cost accounting work?

    - *Digitally – please list the software systems used (if any)*
      - *Single integrated system*
      - *Multiple independent systems*
    - *Manually*
    - *Both – please specify what is done manually and what is digital*

11. How often does the team meet to review the costing/billing/financial reporting information? (Monthly, Quarterly, Yearly). Please elaborate.

12. What do you see as the main benefits of regularly collating cost and financial information for your organization?

13. What are the challenges do you face in reporting cost information/ financial reporting using these internal management tools? (e.g. Lack of IT support or any other legal issues) What are possible challenges/obstacles in relation to maintaining standard cost information (accounting) practices at your facility? Are there any problems specific to a particular type of speciality as well? (Awareness, Data system, Capacity building, Increase in workload, Human resource & IT system)

Hints:<br>1. Have you made any adaptions to these methods to overcome these challenges and if yes, can you tell us about that in detail?<br>2. How could the methods be improved? How would this help? And how feasible is this?<br>3. How can it be implemented without increasing the work overload of the staff?

14. **(OPTIONAL)** Can you give an example(s) where using cost information has helped/helps in decision-making in the hospital?

15. How would you improve the current costing/ financial reporting/ billing system in your organisation?
    (Hints: methodology, regulation, systems, implementation, software)

16. What do you think about having a centrally provided guidelines for maintaining financial/cost information to help with understanding returns and investment and overall efficiency of the organisation?

Part III: Interview tool for non-DRG providers only:

1. Did you receive any intimation to join the DRG pilot from the National Health Authority?
2. What was your response?
3. If no, what were your key concerns to not join the DRG Pilot?

Part IV: Survey for healthcare providers only:*

***This was administered as a google form. A link was provides to the healthcare providers after the interview and they were to submit later whenever ready.**

Objectives of the healthcare provider interviews

- To understand cost-accounting practices, the role of cost accounting in hospital management, and the capacity for cost accounting in hospitals (public/private/empanelled) in India.
- To understand provider experiences of reporting for the DRG pilot using the PMJAY transaction management system

1. Name

2. Organization

3. Designation

4. Roles and Responsibilities

5. Roles and responsibilities in relation to the DRG pilot (For -DRG pilot facilities only)

6. Facility characteristics

- Ownership: Private, Trust/NGO/Not for profit, Public
- Level of care: Primary, District, Tertiary, Other (Please specify)
- Specialties covered
- Number of beds
- Number of admissions per year
- Number of out-patient visits per year
- Location: rural/urban; city tier; metro/non-metro
- Proportion of patients that are PMJAY amongst total patient load

7. How have you found support from the NHA/SHA and their partners in the rollout? (e.g. Sensitisation; Training; Ongoing support; Any other)

- Very helpful throughout the process
- Helpful most of the time/in most areas
- Helpful some of the time/in some areas
- Somewhat helpful
- Not helpful at all

8. Please can you tell us if the reporting system for the new data fields (software/templates) is easily integrated into your existing systems?

- Very easily integrated

- Mostly easily integrated
- Easily integrated
- Mostly challenging to integrate
- Very challenging to integrate the new data fields

9. Please rate the level of difficulty faced while entering the DRG data in the TMS:

I. Drugs

- Very easy
- Easy
- Neutral
- Difficult
- Very difficult
- Not applicable

II. Consumables

- Very easy
- Easy
- Neutral
- Difficult
- Very difficult
- Not applicable

III. Diagnostics

- Very easy
- Easy
- Neutral
- Difficult
- Very difficult
- Not applicable

IV. Implants

- Very easy
- Easy
- Neutral
- Difficult
- Very difficult
- Not applicable

****
