# Supplementary material for: Establishing national hospital costing systems: insights from the qualitative assessment of cost surveillance pilot in Indian hospitals
Source: BMJ Open. 2024 Sep 10;14(9):e082965. doi: 10.1136/bmjopen-2023-082965 (PMC11409349; doi:10.1136/bmjopen-2023-082965)
Supplement: online supplemental file 3 [file bmjopen-14-9-s003.pdf]

Annexure 3: List of Quotes

Table of Contents

1. **Quotes from the interview with National Health Authority, India**
2. **Quotes from the interview with State Health Authorities of Indian states of Kerala, Haryana, Chhattisgarh**
3. **Quotes from the interview with sampled hospital providers**

Quotes from the interview with National Health Authority, India

	All Themes	Quotes/excerpts
1.	**Sampling of the states**	Geographical, of course, the one factor was of course, geography, and in addition to that, if we see, see, we are being closely walking along with the development partners. Right. So, we have also considered the presence of our development partners in particular states, so that you know we can get the exact handholding sitting in those places. In regards to say Maharashtra, specifically, we have chosen the State because that was a non TNMS state. So, they are not using our TMS. So, we also wanted to understand the aspects of state which is being using their own Transaction Management. Oh, see, 5 is a reasonable figure, I would say so that we will have a coverage of all the different set of hospitals in a way accredited versus non-accredited versus the small, big everything and taking care of small to medium level, of tier wise city. So, with this selection of 5 hospital, we were able to collect the data of all the 5 tiers I mean all 3 tiers.
1a	**Criteria for selecting hospitals**	So, for every state we have tried taking the public and private hospitals. Then, even in the public hospitals, the teaching, hospitals, medical colleges, and maybe the trust and non-trust kind of hospitals. So, the breakup of all the hospital bases on their ownership. So, all specialities, along with the accreditations. So those were the 2 prerequisites of selecting any hospital. After that we tried dividing into the public and private, and under the private and public. We divided it further into the tier wise, as well as the number of beds, so we divided it into the 3 categories of beds which one is less than 50 bedded than 50 to 300 bedded and 200 and beyond. So, 3 levels of the bed strength were being taken.
2.	**Multiple levels of agreement for Pilot**	So first the we received the physical concurrence from the States, the next level of the concurrence was taken from the hospitals so after the selection of these hospitalsIt was not just a even a single round of sensitization so we ensure that they say SHA understood, the hospitals understood, and they were able to, you know, come back to us saying that we still have some queries before we sign the consent form. So, we had that level of engagement with the hospital so they understood that.
2a.	**Dropout rates**	once those hospitals were selected so approximately, we tried selecting somewhere between 20 to 25 hospital per state. So, after the selection of 25, it was expected, at least the 50% would join as the DRG Pilot. So, this was the target. At least 50% should join in, and accordingly we took 20 So hospitals from every State, and after that we shared a consent with all the hospitals and basis, on the consent what they have provided us we have like shortlist those hospitals.
3.	**Resistance to join the Pilot**	So one was, what to so one was their capacity in terms of the main part. So, what they feel is the workload will increase. So that was the one, and the second was, of course, they were resistant in sharing their cost data
4.	**Time frame for Pilot and Possible reasons for delay**	We started the initial phase in the in one go, but in terms of the cost data collection that we are now doing it in the phase manner we have already started in Haryana, in last one week and the next state we will be taking up maybe and then add the 10-15 days' time period. Basically, we've been waiting for our IT to integrate with TMS in the hospitals. So we would first like to reduce their little bit of burden. We thought of taking it into the few hospitals, because our TMS usually do all the implementation based on the State there is no individual hospital wise data integration is possible so that was it was a bit difficult and a different task for our IT to do so, doing it in a hospital way wise, manual in a selected hospital. So, it was a time-consuming activity. we may extend it to another 3 months or so we were, waiting for the DRG groupers, which was supposed to be provided from the WHO. So, by the time they are working on the groupers, we simultaneously wanted to collect the data, so I think we are bit delayed, but not very much.
5.	**Support provided by NHA**	So, we started with the sensitization sessions. So, the first sensitization we took for the entire you could say the nation. So, all the SHA. Members, all the State members, their CEO's, and everybody, was been involved. we have also formed a technical committee. So, we have a technical advisory committee. We have where we have the multiple international experts and the development partners are also part of that. So along with that, we have decided upon the pilot states. And basis on that pilot States. Then we gone onto the specific sensitization sessions. So, first session was like taken for the entire state. Then bases on that entire state, The chosen hospitals were taken into a next round, and then maybe the third said sensitization was taken in a physical manner, or maybe the visits from the SHA members or visit from NHA people. so

		multiple layers of sensitization took place so those were in the form of presentations, in the form of meetings, the normal discussions.
6.	**Mode of data collection**	As I said, we have integrated those cost fields in our TMS itself. So as soon as the patient is getting discharged, they have to fill this data within the TMS itself. even in case of the in the state and the normal course also, the data is being available at NHA. So, there is nothing in between SHA has to do. SHA'S role comes when the claims payment is considered or the approval of the cases is being asked.<br>So, at that level the SHA has their roles to play, but the data at any point of time is available to the NHA, so even in this scenario the data will be there in the systems which is accessible to NHA and wherever the SHA you also have the access to all those data where they have their data houses or the people available in their IT to extract the data, they will also have access to this data.
7.	**Plans for Data analysis**	we have only added 4 fields in our TMS to collect the cost data since, or since our TMS is being built up in such a way that the data is being collected or you can say the information is being gathered in the TMS in the form of a bundled payment right, so they usually select a package, and everything is being within that package. we will be collecting only the data on these 4 fronts and per patient. So, whatever be the patient, entry has been taking place, so we have not touched any of the parts of the TMS. Except this addition of the data. So, whenever the patient is about to discharge basis on that discharge information, they, the hospital person has to fill in these data of the cost in these 4 segments, they the drugs and the call or the consumables and the implants and diagnostics so they'll be putting up their additional data there and rest of the flow process will remain as it is, so it is to the NHA and the technical team that how we want to add this data, we can extract the data and basis on what kind of procedure has been done for the patient we can take it procedure wise we can also take it per package wise we can take it.
8.	**Monitoring and supervision**	So that can go and see the level of quality and completeness of the data which has been provided by them, different levels of monitoring. So, we have a division in NHA that is monitoring and evaluation. So, that is one division. Second, we have a NAFU, which is a National Anti-Fraud Unit. And the similar Antifraud unit is also being developed in each of the States which is called the State Anti-Fraud Unit. So, all the all the monitoring is being done basis on those the data we get from the monitoring and evaluation time as well as the that is in compulsory audit which needs to be done from the NAFU and the SAFU, so national and State Anti-Fraud Unit. So now, for instance, they have to do the compulsory audits and based on those orders, then we also have the team which is available to do the AI based monitoring as well so in case if they feel that there are some documentation related issues or whether the documents submitted are not appropriate. So, all those things are being checked, and those we would say that triggers is being raised to the NAFU, and SAFU. And then they physically, as well as virtually they check those cases, and the monitoring is being done. And along with that we have a monitoring and evaluation team. So, the regular data analysis is also being done from their side and basis on that, wherever they see that any outliers are there or any major issues are been coming up which has been raised to the concerns team. The first level of monitoring will take place when we have the person available in that particular state or within the DRG pilot
9.	**Challenges:**	
	**Making them understand regarding DRG**	About the minor challenges, yes, of course, consent from the hospitals, and convincing them, making them understand the concept of DRG. And of course, getting IT integrated into the TMS systems. all those resources constraint, and we have started getting that feedback from the hospital that yes, it is a time-consuming activity. It takes time and you know the manpower requirement has increased.
	**In reporting correct information**	Yes, so what we are expecting hospitals to provide us the price, what they are paying, the cost. But we are not very sure that could also be another challenge where they might not provide the accurate costing, they may provide us the MRP, the maximum retail price, so at what they are selling, maybe they can or may share that also.
	**Repeated entries for diff systems/schemes**	1. They have their own HMIS systems available, and I'm sure they been doing such kind of entries on a daily basis. So, the availability of the data is not, should not be a problem. The only problem is at the duplication in entries, because once they might have to do the entry at their own HMIS and the secondary they have to do in the TMS maybe but I'm sure even in that scenario they might not be doing the HMIS entries.

		2. These are already there. The names are there. They have to just select out of it. Maybe the nomenclature is different in their HMIS and in TMS
10.	**Roles/ responsibility of hospitals in DRG**	I mean the entire DRG is based upon the costing, unless they will not provide the cost data how we are going to further group it and how we are going to further make this combination of different resources. See the Most of the things are dependent upon the hospitals. They have to code ICD. They have to code. They don't have to code but they have to select the correct HBP and based on the correct HBP at the NHA level. The ICHI port will also get reflected right. So, even if I forget about the ICHI code, even the ICD code they have to fill. the ground level staff is accurately coding, so that is very much different. Upon the end goal, that's how they are been coding.
11.	**Future planning or goals**	Patient-specific information that may vary across the patients of the same disease condition with different levels of severity, or the rest of the other resources, like let's say, human resource, capital equipment, overheads etc. The determinant of the extent of those resources to be used is actually the length of stay. So the longer a patient stays in the hospital, the more he utilizes the capital, the more he utilizes the other. I mean, there are more doctor visits, more nurse visits, etc. So we are, and that lowest data is already captured in our in our TMS. creating the DRG, making the groupers, and, if not at least a group, was in all. But this will also help us in the further you know rationalization of our health benefit packages. What we have, and while collecting those data, we will also know that you know, in different tiers of the city, how we can plan or help benefit packages

Quotes from the interview with State Health Authorities of Indian states of Kerala, Haryana, Chhattisgarh

SNO	Main themes	Sub-themes	Quotes		
			Site-Kerala	Site- Haryana	Site-Chhattisgarh
**1.**	**Roles and responsibility of SHA**	**Enrolling the hospitals in DRG- getting their consent**	our main role was actually, uh, bringing the hospitals on board. So for first rule is actually sensitizing these hospitals and getting, uh, consent written, uh, signed consent from these hospitals with regards the regard to the as pilot. SHA has the major role of bringing on board the all these hospitals because we are the ones who are directly engaging with them, not the NHA, not the world Bank. The SHA is directly engaging with these hospitals to get their delivery of care for the patients.	Out of 18, 15 hospitals gave consent for the DRG Pilot. We had to motivate the hospitals for their involvement in DRG Pilot. We had to constantly assure them as their was no direct benefit for the hospitals and without that they were not ready to join.	See we were provided with a list of hospitals and we had to get their consent to be enrolled in the DRG Pilot.
		**Coordination between hospitals and NHA**		But I think the main involvement was in coordinating between the NHA and the hospitals.	
		**Understanding the challenges hospitals are facing**	recently, last month, again, we had a call with, uh, these institutions regard because some of them have got the, uh, tabs in their TMS. Some of those institutions hasn't got the tab yet. So, we again, collected data from them. What is the challenges they're facing, uh, regards to their, uh, providing the information in the DRG		
**2.**	**Pilot/ Training started**	**Pilot Started**	November, not before, not long before that, maybe in November.	It was rolled out in October or early November, I guess.	But It (DRG) was started in the end of October or in the first week of November.
		**Training started**	We had at least three or four annually, but you can't really call all of them as training sessions, right? We do not have any properly organized training workshop instead, uh, we had, uh, sort of gap filling	It was started I guess in May 2022 or June I guess	In the month of February- March 2022, training sessions of ICD were conducted. For DRG it was in April

			sensitization sessions in between, uh, but not really a lot of, uh, fully organized training sessions.		
**3.**	**Coordination between SHA &providers**	**Coordination**	We also made a WhatsApp group where, uh, many of these, uh, institutions,	No, no coordination. they don't report any data to SHA	It's a combined effort of SHA and WHO. Providers are empanelled with SHA, they have no relation to WHO. WHO provides training . Can't tell much, because we have just started<br>so after taking approvals from sir (SNA) things approval. I coordinate with the providers regarding field visits, capacity building, organising trainings.
		**Sensitization sessions from SHA**		No, we just communicated through one mail and said that these hospitals are selected and all the sensitizations will be provided by the DNO's.	
		**Support to providers**	It's basically based on the need of the provider and, uh, the availability of, uh, our, uh, support staff here who can actually, uh, visit , if required, take a call on, uh, take or discuss with the hospitals. We had a for few rounds of discussion with the hospitals. We, but we, I think we visited especially exclusively for the DRG , uh, pilots in the last week of October and the first week of November, uh, or, um, where we had a few, uh, people from the World Bank coming in, uh, two consultants from Burbank coming in. So we, myself and Dr. Vimal, we visited along with these consultants to, uh, in four or five districts, uh, to some of these DRG Institutions. In fact, uh, we had, uh, yeah, we were in discussion were to provide a support area		

**4.**	**Roles and Responsibilities of development partners involved**	**Coordination and sensitization with private hospitals**	currently they helped us in sensitization of these hospitals. Uh, then we had a, uh, a few rounds of, uh, calls, zoom calls, uh, another meetings in which they also sensitized our district project coordinators.	I was in direct coordination with hospitals and visited the hospitals for multiple training sessions. ICD training and orientation sessions were provided to the hospitals enrolled in DRG.	mainly is the coordination with state, with Private hospital training, and so coordination is one point of one area, you can say. Mm-hmm. Uh, second area is capacity building. Mm-hmm. third area is monitoring that hospitals are working properly on DRG or not.
		**Capacity building**		Any information or training from NHA was provided to SHA then to District IT managers & District coordinators	so after taking approvals from sir (SNA) things approval. I coordinate with the providers regarding field visits, capacity building, organising trainings.
		**Understanding the challenges hospitals are facing**	And, uh, recently, uh, they've been supporting us in understanding the challenges as well		
		**Hospital monitoring and supervision**			
		**None**		I didn't think there is any involvement of GIZ in DRG Pilot. GIZ had provided us those consultants and we could assign duties accordingly.	
**5.**	**Preparations to ensure timely reporting of data**	**Conducted separate sessions**	We conducted a set of ICD trainings with, uh, with the hospitals as well. Because, you know, ICD 11 being a major part of, uh, the DRG information, uh, the hospitals needed to input the ICD 11, uh, codes.		To get the consent sir conducted a separate session on DRG, and he chaired that session and sir informed what is the DRG and what, how it's going to be useful in the long run.
		**Getting rate contracts for public hospitals**		We tried procuring the list of drugs used with their price for the public hospitals but it was not easy. the public hospitals don't have the price of the drugs used, so we said to enter 0.5*9 or 0.5*0 so that when in the need we do data analysis, we know all this was filled by the public hospitals	They are sending letter to get the rate contract. Then these hospitals will get rate contract and then even we will get that rate contract and we will share this with NHA also
		**Field visits**		then when I visited the hospitals, civil hospital Panchkula twice, Medical college, Mullana, twice, with the NHA	

				team, then handholding training sessions were given	
6.	**Checks in place to assure quality of data**	**No Audits/ Feedbacks**		So, ma'am, basically you don't have any checks in place that they don't report you anything and you don't audit the data that they are reporting. there was no retraining of the staff after reviewing the data. Also, there were no check in places. We had a WhatsApp group to solve any issue.	No, because these social media activities can backfire too. If one person talks to another that no I am not filling this and nothing happens then others will also stop. When I will have the data, then I can make the group and say that this field is missing etc. E.g., in one hospital it's a charitable hospital for cancer patients I came to know in field visit that they are filling NA in unit price so I asked them the reason to which they replied that sir we don't have rate contract. So, they are doing what they think is okay.
7.	**Support provided by NHA DRG Pilot implementation**	**Training sessions**	There was sort of sensitization, uh, meetings. during the sensitization sessions of the SHA. Uh, the DRG discussions and initial sensitization sessions and, uh, followed by the online, uh, zoom calls for trainings of these hospitals for training of our, uh, district project coordinators.	So SHA was part of that training. We were in the training sessions. So, it was through NHA and SHA. No IT support but there were training sessions. Yes, we were in the training sessions, First NHA provided trainings to SHA's then we gave some trainings to DNO and hospital's managers.	So, Capacity building training sessions were done then NHA provided ICD 11 & ICHI training sessions in March to SHA. Then ICD was integrated in the system around, Uh, June or July.
		**NHA capacity for training**	So, we visited, uh, a hospital at that point to see the potentiality of, uh, them being part of the DRG pilot. So, a similar visit to understand and explore the hospitals. Um, to, uh, get more hospitals on board.	When we launched this then Dr Shankar did the sensitization of all the hospitals again. Three rounds of training sessions were provided by NHA, Three rounds were provided for ICD training. Then 2 rounds for ABDM	
8.	**Benefits perceived from SHA and healthcare providers**	**No benefits**		No. Even hospitals are saying that we are overburdened, we don't want to be a part of this. Its time consuming. I don't see the benefits till now	
		**Views on implementation**	the DRG pilot obviously can be very helpful, uh, in payment optimization of within the packages, you know, uh, within	It's been 4 years to the scheme and I think we are still in its early phase. We	

			the current things in optimization of, uh, payments, but the information that we capture is, uh, uh, might be a lot incomplete, you know, and if we go at this space, it might require a long time to collect that information, to reach at some valuable, uh, conclusion or some valuable.	are introducing DRG in the early stages, that is what I feel.	
**9.**	**Challenges**	**No benefits and extra manpower**		Hospitals are refusing to take part in this saying that we don't have enough manpower. This is just extra work for them. At present they don't see the benefit and the utilization of this. In future when it more refined and there will be added benefits in packages may be then they will see and accept it.	
		**Training challenges**	in the SHA at the state level did not under, did not, was named, not able to understand that very well. And as an result, when we were training the hospitals online or through Zoom, uh, again, there was this, uh, gap of, uh, communication, gap in communication. So, I wouldn't say there was a very good quality, uh, workshops, even online. There was no workshop that was held, uh, physically with these hospitals. Even the online workshops, uh, were not very well, very properly organized or, uh, attended often also. That was a challenge actually.		
		**No physical workshop**	there was no, uh, not a very well organized, uh, physical workshop or physical workshops. I think that is one of the drawbacks that we faced with these hospitals because, uh, We did not understand, uh, the DRG pilot very well initially.		

		**IT Team**		No IT support or training session was provided.	Whereas here in SHA they have only 2 data analysts and that too on NIC's payroll. They want to see official emails and communication before starting any work and that is time consuming process. I have communicated this to NHA too but they are saying that SHA can do it.
		**Time management**		another problem is doctors at these hospitals are overburdened, they have so many things Doctors too had a session in DRG pilot. But they cant see everything at portal, they don't have that much time. Doctors can't help much because they have so many patients to see, in public hospitals everybody is overburdened.	at the same time they're facing challenges in terms of HR and time management, TMS is also slow.
		**Mindset**	DRG tab is not mandatory, so the hospitals doesn't have a felt need, uh, to take a meeting with us to resolve the issue. Uh, no if, unless they're, they want to enter the data very properly, but we were having this, uh, need and we took the call, uh, last month.		They have realized that they will get the right incentives for the right kind of resources or services they will provide. As of now, since it is pilot, they are, I would say they are involved, but they're not taking it very seriously. It's just Indian culture that until it is implemented, they will not give hundred percent participation either
		**Lack of information with PMAM**	One major challenge, uh, with these public hospitals is that, uh, there is no organized, uh, way of having this information, particularly for each sort of patient. you have, uh, especially the consumables, you know, you, there is no real track of which, how much consumables individually, each patient is consuming, uh, in a hospital, in a, in a major public hospital	The procurement list is not there for public hospitals. So, they need to have the list to fill the price of the drugs or implants. Its hard to procure the list.	Challenge is to get the complete data. DRG data entry is not so tough, anyone can fill it. The point is, you should have the data with you. You should have it in the discharge file, you select medicine and gets a drop down list automatically. But some of the PMAM are not filling the data because of lack of information. Like they don't have information of rate contract. They don't know the unit price of 1

					medicine of paracetamol. So, they are leaving that space empty.
		**Lack of understanding around costing**		At ground Level, PMAM, hospital and other manpower won't be able to understand the need of costing and the intent of DRG. Even higher officials needed time to understand this.	
		**Capacity**	Not just like that, but, uh, no, they, they, those PMMS or these data entry operators will have to be specifically trained to input all these data, and it's not really within the capacity of SHA to go to these big public hospitals or to go to the all these hospitals and train all the individual argument. Some of these responsibilities will have to be taken by the district or the institution itself, you know, if they're, uh, if they're happy to be a part of the pilot. So that is also another challenge with regards to the data entry, uh, of the DRG uh, information.	Even some nodal officers are not clear . See, we were also the part of same sensitization session, it was one and half hour of session and we attended the same session so may be the owner joined the training, nodal officer attended it and the person who is supposed to do it was not part of it. This might be the case. They are so overburdened that they can't focus on everything. There are chirayu cards too. This is my understanding of DRG Pilot. I don't know If I am right	
		**No clear understanding**	not a lot of clarity goes to what is going to be the role and what is going to be additional data that the hospital need to provide. What is it, uh, additional burden with regards to the resources, especially the human resources that the hospitals will have to invest in, or we'll have to assign some responsibilities too to be a part of this, We also did not have that clarity a lot.		
		**Selection of hospitals for Pilot**	Uh, in fact, we did not get the required numbers that we were hoping for. Initially we got early consent from eight, I believe, and then we expanded up to 13 or 14. But still now there are, uh, uh, few, uh, major districts, metropolitan cities, uh, metro		

			cities from where we don't have any hospitals right now, given the consent, like cochin.		
		**Increase in workload**	the, uh, the time that they need to spend the resource that they need to use for inputting the data itself. Collecting the data, and inputting the.	Their workload is increasing, they are not getting any incentive, they were asking that if their claims will be approved at a priority or they will get TMS approval at priority. All the facilities asked for manpower, because PMAM have too much work,	
		**DRG TMS**	The first challenge is that many of them did not have the DRG tab. Uh, it still not, did not get the DRG tab for it, inputting the data. Uh, so, uh, uh, the first challenges regarding the data is that many of these hospitals still not, still not have got the DRG tab in their TMS. And, uh, even if they have it, they, uh, because it's not mandatory and I would not suggest it to be mandatory because a lot of information will be missing.		
		**DRG not a part of original contract**	that is not a part of the original agreement, uh, between the hospital and the SHA itself. The DRG pilot is not part of, uh, the original agreement. So in that case, this is becomes challenges when we are not able to fulfil the paying, uh, the payment, uh, terms, uh, on time.		
		**Data Sharing**	with regards to the requirement of, uh, you know, uh, the, what is the information that is needs to be shared. Let's say if a hospital shares that, uh, no, they're, uh, spending less on a patient, the hospital, the private hospital will be concerned that, uh,		

			the package rate might be reduced tomorrow because they're seeing that the package uh, the, the cost is not as much in delivering the care to the patient.		
	**PMAM Challenges**	**Keeps changing jobs/departments**		doctors were writing abbreviations. then we asked the interns to write full name rather than the abbreviations. In college there is another issue, rotational trainings, so in OPD, we had to train a batch of students every months because they will shift to another department in a month and then the other students will face the same issue and PMAM wont be able to enter the data and quality will degrade.	Again, the biggest problem is PMAM keeps on changing. We have given so many trainings and we know how many PMAM have left those hospitals in DRG hospitals. So new PMAMs have joined and they have not received the trainings.
		**Educational qualification**		The condition is same at government and private facilities. Ayushman mitra is 10th or 12th pass, they don't have such high understanding. They don't know what is primary and secondary diagnosis. Even salary is so less. I guess it's 5000. when the hospitals joined, they were overburdened, PMAM could not understand the primary diagnosis and secondary diagnosis. Even after so much training their IQ is not that much	
		**Data entry**	main challenge regarding the data entry was that, uh, they needed to input a lot of information regarding	Also we suggested to make the primary diagnosis mandatory otherwise no will fill it. This is not a	

			the medicines. Uh, Uh, investigations and all of those.	problem in medical colleges but all the facilities don't have such coding system.	
		**No understanding of medical terms**		All the facilities asked for manpower, because PMAM have too much work, and they don't understand medical terms.	
	**SHA Challenges**	**HR**	Uh, it's difficult to get these things done with the fewer number of people. That's why we were insisting with the world band team that they come in, be a part of it, so that they can have some free time consult who can actually travel to the district and the hospitals if they're require.		
**10.**	**Suggestions/ recommendations for improvement in the DRG process**	**Access to DRG data to SHAs**	so we, we have not got the access to the entire set of data that has been entered by the hospital still now.So, once we get that information, we are in a position to tell the institutions as to this is the gap that you are having, and then you need to focus and put this data more properly. But we have not got the access to that information yet, uh, from the development team.	No idea, Can I or Can I not. We are so overburdened that, the person who was looking after this is not here, it's been 15 days that she is not here and I don't have time to see. There are so many important parameters to see. Recently they asked us the cost of government hospitals so we sent a mail that this is all the cost for government hospitals.	I have not seen the data yet, so I don't know the actual quality and what they are filling. So, I cannot comment for sure.
		**Incentives**		So, they asked for manpower, or at least salary for a staff as they will need to hire.	There is no incentive for them right now. If we want something from them I think there should be some incentive for them
		**Minimum qualification for PMAM**		These things are successful if you are medico, either a staff nurse, at least minimum should be staff nurse. The hospital had raised this query too,	

				either pay us for an extra staff if you require all this information. at least a person with medical background is needed.	
		**Course work/ specialised individuals for the job**		I think course work is needed. We need people who are trained specially in this and they get certified and they should do this work. At least the person should be graduate and the benefits from the scheme should be clearly communicated to the hospitals. There should be a 6 months course work for such personnel	
		**Non-monetary incentives**		they were asking that if their claims will be approved at a priority or they will get TMS approval at priority.	
		**Manpower**		we should hire a person with at least a medical background or someone who has a basic understanding of this	
		**Consolidated template for the information**	uh, we needed to have a cons place where the information is consolidated. For that purpose, we requested the World Bank and NHA, they provide a format.		
		**Gap analysis of the data collected**	regards to the, uh, data entry that needs to be done or some sort of gap analysis that needs to be done. So, we were insisting that some person be assigned full-time so that this activity could be done.		

		**Include more number of hospitals step by step**	let's say, uh, an eye hospital or, uh, who has a reasonably well, uh, in-house information system, management information system, or another private hospital or a smaller public hospital, uh, and then go on and expand the number of hospitals on board in this DRG pilot, it might actually make more. Rather than trying to get, uh, 15 and 20 hospitals in one go and, uh, failing as we go instead that, uh, no succeeding in each step as we go. Take smaller steps, but succeeding each step as we go		
		**Capture easy to collect data first**	Capture data that is easier to capture with, and then build upon the, the, uh, data requirement from these institutions, you, whatever is easier to capture. Let's say the investigations are easier to capture or the medicines are easier to capture, and then step by step, if you can do it, it might be easier, uh, to get, uh, these hospitals because then we'll also be in a position to give some answers if there's some queries		
		**ABDM complaint HMIS system**	use some sort of MIS that is compliant with the ABDM. if there is some sort of such, uh, you know, policies in place that the private hospitals has to use some sort of, uh, MIS system that is compliant with the nationally set of set of policies, it is easier for data to		

			be captured across systems know, uh, at any point in time you can actually capture what is a required number of, uh, what is it, how many patients are there in each hospital. It's also useful for ABPMJAY, it's also useful for HMIS, uh, general Health Information, health Management Information System, HMIS as we call it, where we actually capture, uh, almost, what do you say, more than hundred, uh, 200 indicators, uh, across, uh, the hospital and the field performance.		
**11.**	**Emerging themes**	**Facilitating factor of DRG pilot**			one thing about the DRG template is it is very easy to fill, you don't need special skills, anyone who has ever worked on the computer can fill it easily.
		**DRG Pilot hospitals selection criteria**	Um, so we had around, uh, listed from five or six districts around the 15 institutions, uh, which were potential, uh, institutions including both public and private. So we looked at a mix of, uh, both, uh, the big, uh, institutions like medical colleges and also some specialty institutions like the eye hospitals and all, which already had a good, uh, system in place for, uh, for their internal data collection tool , 33% would be public and roughly one third would be public and, uh, two thirds privately.	1. the Bed strength of the hospital: more than 50 beds or less than 50 beds.<br>2. Then we needed the hospitals that had some cases, so in coordination with the TMS, we selected hospitals with more cases because there was no point selecting the hospitals that had less number of cases.<br>3. Then according to the criteria, we needed some public and private hospitals<br>4. We also selected two onco-specialist hospitals	

		**Reporting for DRG- Private sector**	They give you a bill for, let's say you have, you are doing your stay at the hospital. Uh, X 16 gauge canula have been used three times or. Uh, the seven and a half size gloves has been used X times. Uh, other gloves have been used so many times, so they were, they already have the information, uh, system to capture the consumables use within the hospitals anyway, and that is essentially the information that we require in DRG pilot.		
		**Reporting for DRG- Public sector**	But, uh, we are looking at the potential of, at least in enrolling more, uh, hospitals, even if it is a single specialty hospital, uh, into the DRG pilot, especially private ones. Uh, that is also because the public ones, uh, doesn't have information capturing mechanism within their institutions itself. No. I always tell my from a private hospital, They give you a list of all the things that you have used in a private hospital especially essentially that is the only information that is required for a DRG pilot anyway		
		**Cost data availability- Public**	The problem is, uh, that historically there has not been a track of consumables. Unless it is a consumable that is not available within a hospital and the patient has to go outside and buy. So, in many		

			public hospitals, uh, either the hospital is, you know, giving these smaller, uh, elements for free, or the patient has to go out and buy it. So, there is no proper track of many of these consumables, uh, within a public hospital, let's say, uh, x number of, uh, so and so consumables has been sent in bulk to a particular ward of this hospital, say ward number three of this hospital. And that is spent on these patients, uh, as and how it is required. And especially on what, uh, the patient in bed number one consumed as to patient in bed. Number two is consumed as not real properly tracked, documented anywhere in some public hospitals maybe, but in most public hospitals not.		
		**Cost data availability-Private**	Many of these hospitals do have it because at least in some form, they do maintain, uh, these lists because they have to ultimately build the patients anyway. Oh, no. Unless it is our, uh, it is the ABPMJAY beneficiary where, uh, you cannot bill the patient, but you still have a, uh, information that is tracked regarding the consumables and the investigations and the medicines that were spent on each patient.		

		**Data availablity**	the chief medical officer of the district or the district medical officer, decides to review as the, uh, programs, uh, or the national programs or the district's performance with regards to some health, uh, information, health, uh, indicator. The data from private sector is missing in, uh, most of these parts. That is also because there is no proper way of integrating, uh, the data that the hospitals have. Uh, we in their mis or we in some form with the HMIS or with the nationally required set of indicators.		

Quotes from the interview with sampled hospital providers

Indent	
**Facility number**	**Colour code**
Facility 1	Dark Red
Facility 2	Red
Facility 3	Green
Facility 4	Purple
Facility 5	Lavender
Facility 6	Yellow
Facility 7	Blue

SNO	Main themes	Sub themes	Site I-Kerala	Site II- Hayana	Site III- Chhattisgarh
1.	**Orientation and Training**	**Orientation**	Then when it is connected to DRGs coming at pilot, they go training from district level. District level offices come to our institution and they go training to them. And whenever they, that is, whenever they need training. We also, uh, our PRO is go to their, uh, counter and they, she also helps them and, uh, through on phone, uh, connected to district level. And then they got online training from the, from that. Four to five sessions held by the NHA for the orientation and the initial training for the D R G pilot for the hospital. Uh, uh, about four to five virtual meetings were contacted, uh, and later on, uh, all the support currently is being given.	Yes, NHA provided us with some online sessions. But that was not beneficial, because that was advance for us and we don't have any basic understanding of the topic. They were telling us to maintain records like this and that but in reality, we have staff shortage and the dealing clerks have to maintain records in n number of ways. This is very difficult and we have to rely on PMAM to maintain the documents and then give it to accountants. I don't remember exactly maybe we would have got a session as well but we received some documents on to which they wanted our consent. We received one online training	Online training was given during the meeting, some information was given. Related to DRG only one session was conducted and one physical. Yes, we got one VC was conducted but after that what happened we don't know.
			the medicine bill, lab bill, and all because we are not trained because partially only, we can	Yes Ma'am. Training was offline. Maybe I don't remember now. So, there is	I was told about that training yes, but I was not able to attend the complete and I don't remember

			be able to upload the details of the pages. they had one training that was done virtually. We got, we got training programs conducted by, conducted by SHA (State health agency Kerala) and also national Health Authority. He's (the staff responsible for entering data) very satisfied with that training program. I think, uh, two or three training sessions only. We are getting, uh, this staff only attending two only. Yes, we are satisfied. we have all the training. You have all the training, They received one training from the NHA regarding this ICD and data entry. And then it was like the DPC who handled them, uh, regarding this. So, all their queries and, uh, difficulties were addressed by the district coordinator. There has been a WhatsApp group which is created, uh, for the hospitals running DRG with the SHA personnel. And I think the	no problem in that. Everything is understandable. No, there is no problem in understanding and also in working. Yes ICD training was done earlier but nothing as such comes because till now ICD itself selects the package. There is not much role of it. Earlier we did not understand anything but now with time we have learned the system. We enter pathology, drugs, consumables (pre formed list) along with implant and diagnostic tests. Only one session was there. ICD training- Yes, information was provided for both and everything was understandable in detail. We are satisfied with the training as well. ICD training was done separately. No. there were no trainings. There was just a format that we were given and we had to fill it. We just received an email that this is a platform and all this information is needed. There	that completely. Two training sessions for ICD and two for DRG. For ICD I attended both but for DRG I attended only once. Sir came for the physical training. 2 training sessions were conducted: 1 before the start of DRG and 1 when DRG was started. Training regarding ICD was given related to patient code extraction in the starting. DRG related trainings have not been conducted. We didn't attend the orientation session as well. No Training, I will be very honest, that is not sufficient. If the program is being launched on a Saturday if I remember. I called for training, Monday or Tuesday they had arranged the training. But that was not enough. We were not informed regarding ICD training. Actually, one online training was done. And one for ICD coding both done online. in ICD we do not require more training. it is easy. If we have any doubts in description, then we can also ask from doctor.

			NHA personnel is also there in the group, I think, and that is how they are getting their issues resolved currently.	were 2/3 online meetings and the issues that we were raising were not addressed. Both online and offline training - Earlier, we were unable to host the DRG TMS on our system. So maam from Delhi came and checked that.	
		**Overall process of entering data to the Transaction Management System (TMS)**	Madam, uh, initially I told you that is initially that the doctor should complete the case sheet. Mm-hmm. Then, uh, they are collecting data from the case sheet. First step on, then after data, that is after they are to enter the data of investigation. Likewise, investigation, uh, report is also, uh, that that data is getting from the bill, bill. Investigation, uh, that is getting from Bills. 2. Already every data is come here. This. DRG. The DRG formula. Secondly, lastly, one DRG form. We have to enter in that form. We have to the data, additional data, in that okay. But are available from the E billing. E billing. additional	portal doesn't have option for day wise entry. So, we enter at once. In case of medicine department, the number of medicines is high. initially when we were not very clear then we used to write 0, because we are not procuring the medicine. If we do, we write MRP. At the time of registration. Its compulsory. The procedure is bit different for government and private hospitals. Private hospitals have to take permission before the procedure. We have to provide treatment in any case. So, we will submit but still do the procedure.	We receive the file and at the time of discharge data is entered, in the system bill is prepared under which record of medicines and consumables are maintained and then filled in the DRG accordingly for every patient. This is the format under which price of the drug, quantity is mentioned. In the bill, price is also mentioned, it has all consumables that have been consumed. Not day wise but at the time of discharge. If the patient is discharged on the same day, then data is entered on the same day. During discharge, nurses come to us with the file of the patient and then we take the thumb impression of those and then we discharge them in one day only. We take the finger print and save the data on the site only. A page comes named as Case

			data means me, medicine, and, uh, diagnostic. That data always written Sir, uh, that bill is zero. Medicine is available from here, so it is zero. Consumable consumes, uh, in DRG. That is that one point is consumables, consuming using from here, this institution itself. So, data also shows zero. Everything is through e-billing. Okay. 2. whatever the high-end medicine or high-end investigations which is being done for patients that is being entered as per the rates available at the hospital. the patient needs to be discharged, uh, but they have to update the discharge in the TMS, and that is when the patient is discharged in the, uh, from the hospital.		sheet and treatment into which we fill the details and take the finger print as well and save that. So that process is saved till there and whenever we open that page again it will come from there only and after that we complete the further process. Firstly, we complete fill the DRG entry, then it also required for thumb expression once you complete fill the DRG and submit then again, we cannot open and it shows the claim. So, when you block a package and actual process is started you need a bio-auth right now the government has made it mandatory that while a patient is being discharged you need to have a finger print. Now we can't just take the fingerprint and say the patient that you can leave and then we will the discharge because ultimately you have to fill the details then the option of bio-auth comes.
2.	**Sources and quality of data**	**Sources of data for entering into the TMS**	4 data entry operators. they are working 24 hours. Qualified with the data entry capacity. That is computer. Computer knowledge is for them and they are taking. Um,		We are three here and five new persons has come. These days patient load is increased so new person have been appointed. So whenever there training will be completed the workload will

			data entry operators are specifically for PMJAY. staff from the hospital, not additional.		decrease. when we realised system is very slow. Then we increased the system and staff.
			Case sheet is main. that is, uh, our madam. Our hospital is national standard. doctor he signed. One data is, uh, getting from the cash sheet and also from the e-bill e-billing. We are maintaining a sheet of Pharmacy sheet and consumable sheets, every consumable we are taking, injections patients are taking for a particular patient. We have a list for with every patient, with the name of the patients, separate sheet are there.<br>Uh, with respect to the, uh, files, uh, for. Uh, with respect to the, uh, files, uh, for each patients, we are keeping, uh, from, uh, from the time of admission until the discharge time. We have our hospital information, software we are collecting from the digital.<br>DRG data entry only thing is	Most probably, it is to be entered from file. Case sheet maam, it's all there. It's in the nurse's chart too. so we enter the price which has been charged to the patient in the bill which is a discounted price. drugs, implants, consumables etc are entered from the case sheet of the patient. We use the bill generated from pharmacy for drugs and consumables. We have lab for diagnostics. We use our hospital HMIS and the bill generated for entering DRG data. From Magnet and bill that we have generated	Okay so you fill the details from the bills only. Just to re check if I don't understand something from the billing sheet. I have both files (billing and case sheet) with me. all the district hospitals/government hospitals get medicines, consumables and implants from Chhattisgarh medical corporation and they have their rate contract. We are doing it according to the hospital as per we receive those lists from the wards (as per usage there), and also for some tests we have the knowledge regarding rates of the tests like CBC so we put them accordingly. Quantity we get to know by the case sheets of the patients. We put as per our knowledge like according to the number of injections given, we write the number of syringes used. From the notes, if OT notes are there, we check for implants. We get the data from sisters (Nurses-case sheets). We have made an

			that they have to enter the rates of the, you know, uh, drug and diagnostics, which is being done at the hospital. But, uh, like he said, it is readily available at the hospital. So they have a hospital software, uh, basically their own version of the HMIS system, which is available, and they have the cost, I think they have, um, they're getting the cost details from the software system. their hospital HIMS system is commonly used for all the patient respective. they're saying that all this rates and the drugs and consumables, which is given to the patient in the investigation, the rate, all the bills are already available in the case sheet. So now they just have to enter that into the system. the drugs detail now. Primary Diagnosis and secondary diagnosis.		excel sheet under which everything is mentioned by them regarding consumables like syringes, masks and gloves etc which copy paste in out sheets. manually that could be taken from pharmacy but we don't have a software into which we put the name of the medicine that automatically calculates the rate of that medicine. Our account department do have that, and they help us out in filling this information. From IPD notes and file, from the case summary. We have made a separate billing excel sheet for consumables of OT and ward for this and also for my costing which I am doing. I am also doing costing for per surgery for example MRI I want cost to be done for my organization so that is my perspective. One sheet helps them and the other for me if I want to look at the cost per surgery. Actually, we have a file which one sheet have all the procedure are enter there or on daily bases tests noted. We cross check using patient's billing or investigations reports. Sometimes we get the

					reports on phone. Or sometime we do not get the report. Then we carry Forword the patient payment slip regarding to get the missing report.
		**Quality assurance and auditing of the cost surveillance data**	The dialysis cases are being entered directly into the system, but all the other cases are being reviewed, uh, are entered only after being reviewed by the nursing superintendent of the hospital. That is by monitoring Madam. We are authorized as uh, one clerk and then PRO is monitoring this always our PRO public relation officer of NHM. She's always monitoring there. That one. Okay. Okay. But first step upon that one. So, in another one is, uh, there is no, uh, means difficulty rising from pupil that is from beneficiary. They are not complaining anything about our services. So likewise, we are, we are the team monitor that one. 3. Madam Monthly, every monthly there is a meeting at	No, there is no team. It's a pilot project that's why there is no checking. I enter when I am sure about the data. In case I have doubt I ask my senior or the doctor. We use the data from pharmacy and before it is released, we check that billing information with the prescription, so that is how we ensure quality.	1st we enter from bills so we cross checks the data and second is from the medication chart. We keep records from patient file as well by scanning that and on the basis of that we enter the data. I do check that, like I do for Ayushman as well as we have a nodal officer who regularly checks the data entry done by these people regarding the quantity of the medicines entered.

			a district level meeting is the. Every Monday. Okay. That is, uh, uh, if there is any mistake from our part, the, uh, that is rejection arises, uh, somehow in our hospital, I think four, three months, there is no rejection. Whatever we are entering is correct, because we are entering only partial data but other things, we cannot able to enter because. We have Pharmacy sheet, lab sheets and there is separate sheet? Actually, in our, in our organization, we have got a quality department, especially for that. They're doing, uh, every month they're doing quality check of that. quality team who do a random quality check on a monthly basis, ma'am. they are saying that they do not face much issues regarding this quality assurance problems or the ICD data entries. They don't have any specific quality check mechanism before that entry. Okay		

			So, they are doing regular auditing. Actually, we are auditing from SHO, they, they're coming, uh, I think twice in a month. They used to come to least my organization, and they're doing the auditing.	No, quality check.	Not yet. No, one comes if face any kind of issue then I contact Suneet sir only. No, audits from SHA/NHA, we check the data from reports and investigation's report.
3.	**Challenges faced by providers**	**Challenges faced during working on the TMS**	That is that work there work is completed within 15 minutes. If sometimes there is site is combined like, uh, arises at that time, more time is taken for completing, uh, entering this. Okay. Time is only the problem. 2. Site is slow madam? So, they have to take more time for entering the data regarding patient, but sometimes patient will come queue in queue. That is the only problem. 3. While uploading the documents in your portal. We are getting some issues in that portal. Okay. Taking 20 to 30 minutes for single, single data upload. 4. because of the portal issue, we are not able to upload our documents properly. 5. TMS	It's slow maam, when there is high patient load, then its difficult. Sometimes the page expires and we have to re-enter the data. It is very time consuming as we have to every time one by one and then we save and move to next. It is very time consuming. once in 10-15 days, there is an issue that the system works very slow. Otherwise, there is improvement in the system. Earlier it was very very slow.	For DRG, TMS is fine. If long time is taken then the session expires and we have to reload the page. System is okay like sometimes there is a network problem but nothing else. TMS is very slow, there is always one msg in group. Facing problem in registration, in TMS. when we realised system is very slow. Then we increased the system and staff. TMS is slow. Suppose in a patient 4 types of drugs are given and they have entered 1 medicine and put multiple in the field. In formulation also we have done it multiple I mean is it an issue of software or properly accurate name of drug should be there. Sometimes, there is no name of the diagnosis in TMS, so we just enter and whatever option shows in TMS we select that one.

			portal is expiring within five minutes. Uh, okay. And, uh, this is, uh, this is making, uh, more, uh, time consuming. 6. The overall speed of the TMS portal is very bad actually. And uh, also we need to, uh, actually in the DRG window, uh, dropdown list is, uh, actually our medicine, which is given to the patients and all other things. And, uh, diagnostics are not, uh, available in the dropdown list actually. 7. Sometimes, uh, the data are. Uh, getting loss, uh, uh, when, when, uh, around the three or five minutes we are not using portal, uh, okay. In making, uh, automatically expiring and logging out. the system problem system is delayed most of the time and the system doesn't respond, respond most of the time, which is also delaying the discharge process for the patient. the system is fast enough. They, the, you know,		

			that entry process can also be expedited, like, um, that is the major part, which is delaying the patient		
			Also, we need, as my boss told, people are coming and going. So we are facing the difficulties in the training them, and he's my boss, told he's my permanent staff. Okay. So one more training regarding these thing. Second thing, uh, is that they had to hire additional four staff because the number of patients, uh, is more at the hospitals. Uh, for PMJAY, they had to hire four additional staff to do this DRG entry. most of the time these are like non-medical pay data entry operators, which the hospital has higher, they're finding it difficult to train their, um, staff to get the proper,	I didn't understand why they were selected; they have shortage of staff, they can't even do TMS, and BIS work properly, how can they do this extra work. If they can provide services only that is also good. we have also shared this feedback to the sha and we want to deliver quality work so we need more staff to do this work.	We recruited 2 persons. We had a new person for only the Mitra thing, so we told him to work for half time on discharge, focus only on registration
			He's basically he's saying that you need a similar provision. Like you know what, right now all the mandatory documents for the PMJAY scheme is being	some help of the nurse is required like I was having the work of heart patients, so in that case ECG is there in file, we count how many times	but the thing is sometimes patients take LAMA and it is difficult to take their thumb impression. It is an additional expenditure on our hospital as well as on the operator.

			scanned. The details the clinical details are not being entered into the system which is ask the documents are scanned. So is asking a similar provision where in these bills or whatever is being generated, they can scan it and okay. They have been told that they have to enter the generic name of the drug which is given to the patient, but that is not available at the hospital. Usually, uh, you know, at that. That is the time when they're collating or compiling all the data and the rates, like what has been spent for the patient. So, when the number of patients increase for discharge for a particular day, then the delay gets more. sometimes she don't understand uh, what is the drugs given by the doctor? What is return in the case sheet or the, so, uh, we, we checking it with the treating doctor or the pharmacy, or we have, it takes some time and	ECG has been done and if ECHO has been done, then report is there in file or not. Angiography is included in the package, if it is there. And other CBC reports are there in which we enter as they are counted accordingly. It doesn't matter if they trained or not, they have to understand medical terms now, visit different staff nurse in case of doubts. So, these are the issues. Main Problem- Receiving of mails from different point of contacts offices like NIA, Civil hospital, sometimes from DC office). Nobody has any idea regarding DRG pilot,	On one side we have data for clinical part, for finance we have another data set and then we compile the whole. Se the problem we have is we don't have a bill counter hospital so nobody has the information regarding rates like who is doing what. we put invoice of devices used. But we purchase those devices in bulk once you have put this invoice number, and after reimbursed it (System) does not accept that. Difficult to calculate per patient doses as well difficult in collation of data from different departments like different oxygen consumption varies patient to patient as well as number of oxygen cylinders changed per day. Different patients using different levels, some are using prongs, some are using masks, some are using for few hours and others continuously. So, it's hard to calculate per patient level usage. So, we calculate an average of 2lakh per month consumption. It's very difficult to get per bed. So, we calculate the average. Total monthly spend in ICU by Ayushman patients

			that is basically delaying the patient's discharge. difficult in adding the drugs required for the patient, the drugs and the lab, which is already available in the case sheet and the rates mm-hmm, but they individually, um, discuss with the pharmacy or the lab to identify, to know what is the rates for the particular investigation or the drugs.		in pharmacy and we pay that. It's very simple way. I think if you also look at the facility level how they have actually planned it we have to sit, have meetings or how we proceed. For us it was not much challenging because we were anyway doing the costing exercise and I was doing it. So, we already have the price list but for other institutes I don't know how is that working.
			It is basically delaying the patient's discharge that is one thing. Because this DRG entry is given at the discharge time of the patient, it is actually delaying the discharge of the patient Very much so. which is basically delaying the patient's discharge because they cannot discharge the patient without doing this DRG entry.	No, because we take their thumb impression and discharge them.	Due to this the work load is increased and wastage of time is there. Every treatment done is already mentioned in case sheet of the patient and medicine chart, and billing. Sometimes server issue is there so discharge also gets delayed. Yes it affects, the issue is we cannot discharge the patient before filling the DRG and for that patient has to wait for even 1 day as well. at the time of patient discharge it is mandatory, so that's reason our patient discharge process is also delay. So linked to the TMS and you can't discharge the patient without this becomes a problem. So sometimes what

					happens is for one patient it takes around 2-3 hours for the discharge. It is difficult for the patient because he has been said in the morning that he will be discharged today but he could not discharge. if we enter correct data then the discharge of a patient will take time
			So, uh, only thing is that they were not used to this concept, uh, earlier. So, the initial couple of months were difficult, like understanding how to do the data correct coding system. But they are now very familiar with the ICD coding entry. They're not facing any problems there. 2. they had little trouble during the initial period on entering, I mean, identifying the appropriate codes. But what they do is like they review the codes with their treating doctor before submitting. So that is what the data entry person is doing at the hospital and also they have received the training for the ICD.	Our diagnosis do not match to those in the ICD Coding. So we put unspecified. In Ayushman, earlier they said that even if you put unspecified, we will reimburse, but eventually when we put the claim, we dont get any reimbursement. Here we don't have IT people. This is our inhouse people who are managing the work as this is a private hospital. And in private sector, they will go for cost saving. We don't have that much trained manpower and we have our inhouse people and they have been just trained by working on the system and as we start getting feedback from SHA, then they take their next steps and further activities.	See she is a non -medical person and operating this system and doing the entry she would not be able to enter the exact dosage of the medicine as a medical person can do. Syrup components do not match, brand name option is not there, we have just components name there, some medicine name we cannot find. Everything covered under the PMJAY. If bill comes diapers are also included in that, things like syringe, mask, banded, gloves, cotton, needle will be there. We are not able to find RN DNS (fluids) as well. Not much challenges in ICD.
			DRG data entry would require at least a half an hour time from the data entry operator to just	It's good if gets removed otherwise only our work has increased. See cost is very	like whatever medicines are there will be entered serial wise only cannot be entered in one go. Due to this the work

			for the day-care patients. she said was the neonatal care for preterm babies who stay in the hospital for two, three months, uh, for those patients and all, it takes a huge lot of time for this data DRG entry to happen. So, um, she does not think that it'll be feasible option for all the hospitals in, uh, you know, it requires	difficult to calculate. We buy in bulk now we have to calculate price per unit for this facility it is next to impossible. Either we divide total bulk medicines bought, it will be difficult. We have that, but its for bulk. Only the store keeper knows how much we received. If we have to then we can but its an extensive work and we need manpower a dedicated staff. Otherwise, its not possible. Everything to be done by hospital, hospital have already shortage of manpower. So Mitra they call it in the program and they are just paid about 5000/5500. And nobody would work in that amount. So, we have our own staff. Because our volumes are very high. The number of beneficiaries is high as in Haryana in the previous 3-4 months they have issued over 30 % more cards.	load is increased and wastage of time is there. nurse is doing that work, they provide us excel sheets of that data. The turnaround time for patients is longer when patients are, they not able to discharge patients. And if you are entering the data of DRG on daily basis they it has to be entered in the TMS and entering in the TMS is itself a challenge especially in the rural area where networks are slow and also every time it gets logged out and they have to re login. So, all of this takes a lot of time. Time it takes to enter depends upon patient stay as well few remains for 4 days few for 10 days. If we will use our Ayushman mitra for this then our routine functioning will be disturbed. Currently, we are not able to get the manpower and our workload is increasing.
			They don't actually understand what is this initiative. the patient needs to be discharged, uh, but they have to update the discharge in the TMS, and that is when the patient is discharged in the, uh, from the hospital. thing is because the data entry	We only require the staff, who can tell us about medical terms. The doctor cannot be made to sit, as he will ask for salary accordingly. We cannot read the files, in the salary which is paid to us. Even if 6 months training is provided, then also we cannot	our hospital provides free of cost services we don't charge patients on the cash counter so we had the data already but how it can be converted into Ayushman that was the problem. So, for that we required another employee to work on specifically that.

			operator is not very aware about the medicines and the drug's part, uh, that is also delaying the entry, but they are not very sure what recommend, what can be recommended in that regard. But it is basically very difficult for them to identify the drug names and, you know, uh, read the doctor's notes and things like that.	read because it's not related to our field. Only this is the issue. Either someone is deputed for duty, who will fill DRG and we will do other file work. So we did according to our hospital. For medicines, it is simple, we have the MRP and we added that. It was sort of mandatory for us that we have to do it	
			for patients especially who has the extended period of hospitalization, the hospital is currently only entering the costliest Drugs and investigation, which is done, they're not able to enter the entire data, which is not practically feasible for them to enter every data for that patient. 2. dialysis because the patients are coming in more frequently and regularly, they are aware. What are the data fields and the things which needs to be entered. Yeah. Their patient needs to be verified.	nobody is able to understand the writing of the doctor for which we have to take help of staff nurse. We don't have staff nurse, we have to get them from Civil hospital. We do not write all the medications or injections given to the patient as its difficult and time consuming. So, we enter accordingly. Also, as we are not from medical background, we don't know the medical terms so we ask our doubts from doctors. TMS asks for the same thing again and again. For example- we have put CBC in the morning, it will again ask in the evening. Same with the reports of histopathology like cancer and PET scan. Requires uploading again and again. It will be same in the evening but the systems	Sir has told for the rate list but we have not received that till now. If we could get that letter then that could be easier for us because without that it is difficult for us to enter the rates. That includes quantity, price. Rarely it works with a slow speed. It is very difficult to enter each and every detail of every single patient can you imagine the work load it is very pathetic to add data in the DRG. It is difficult to calculate by PMAM because syringes change daily, PMO line changes, ryle's tube to be changed 4 times or not even single time so we took average 2 ryle's tube but for medicine it is fixed and for consumables it is difficult. So, we have high number of patients too. ICD is clear.no doubts , but in DRG we face a lots of problem like in drugs ,name difficulty and brands are not same. consumable items are also not

				ask for it repeatedly. For medicines, we have brand names but the options in the TMS have dropdown list of the name of the salt. So, we enter it. The problem is that the number of patients we have is very huge and they stay with us for over 20 days to 1 month. And in one day, there are a minimum of 15-20 medicines and investigations prescribed to one patient in a day. And in this drg, we have to add the data one by one for each medicine or consumable or drug. So, we cannot even discharge a single patient in a day. So ma'am, what we do is, when a patient comes, we enter 1 or 3 medicines of that patient only. We are not entering the complete data, we are entering less number of medicines and consumables, etc as it takes us a lot of time. Our volume of patients is very high. Last month we had 500+ patients. So if we enter every detail of each patient, we will not be able to enter the data even for 2-3 patients	available. TMS is slow, also every individual medicine has to be entered. Every medicine, syringe, gloves everything has to be entered, and we have to enter the data on daily basis. Actually the concern is like we have 100 patients daily on an average so I took the demo of the field and I was asking for the investigation as well. I would enter the data of investigations and medication, strength of the medication and the cost of those 1000 patients is very time taking. How much drug is used. Like in the morning the Dr, has prescribed Amoxycillin TDS and in the evening he has changed the drug then. Another this is we don't have rate contract.
4.		**Benefits**	They already had the system in place through their system	Only our work has increased otherwise there is no advantage.	Not according to me because due to this the work load is increased and

	**Motivation & Barriers to enrolment**		where they were getting the, this data. Um, so nothing like, no additional benefit has come out of the DRG benefit.	Because earlier we use to work in these steps. Register the patient, pre auth him, update the query otherwise discharge him and then claim after it. Few steps have been increased for us because we got stuck as we don't have staff and we have to ask Doctor or nurse what we should enter. It is an additional task. There is a lot of documentation so it takes a lot of time. I don't think no one reads these claims.	wastage of time is there. Every treatment done is already mentioned in case sheet of the patient and medicine chart, and billing. By collecting all this data we can tell anyone regarding how much medicines have been used on the patients. Because we do not charge patients for anything so we had no idea how much we were spending on a single patient. This gives us an idea too. So, for our own organization I don't see that it's not an immediate benefit for us but I think from a public health point of view it's going to be an informative exercise to know what it costs what it takes to do something, so it is from that point of view that it is a useful exercise.
		**Barriers**	Hospitals like medical college would be able to easily do this, but he, is not very sure about the other hospitals being able to do this. Okay. they do not think the, all the hospitals in Kerala will be able to do it	Yes, but it is still in early stage and I think the system will need 6 months to 1 year and after that we can think of feasibility. They should either provide funds to us so that we can hire manpower or send a team on their payroll to collect all the data. They should supervise and monitor them. Otherwise, I don't think they will get the true results that they need.	If doctor will write correct information, then we will be able to do things accurately but till now nothing has been done regarding that.
		**Reasons to deny to be a part of the DRG Pilot**		Yes, we received one mail but at that time it was not clear why they were doing this. So,	

				someone else was handling this unit. So, he refused to be a part of this. What I think hospitals rejected was due to time constraints, we already have so much work and other was lack of knowledge. We understood today what was the main purpose of DRG and why it was started. No one told us the reason behind this initiative. 2. See, what I feel the government wants is they want us to employ our manpower, arrange all this and provide them everything. Research study should not have this motive. I talked to several people at that time, govt hospitals don't have this provision, most of the gov. hospitals work manually plus they have shortage of manpower. We just can't give this work to anyone.	
5.	**Recommendations to improve the process**	**Recommendations/ suggestions**	Provisions if they get a provision to basically upload or scan the bills which is generated for that particular patient, it would make their life so much easier so that is what they're saying. 2. Actually, in the case sheets we are scanning the case. We are not typing n all like that. We are	It is difficult to add one by one, there should be a provision to add everything together and then save and move to next. maam there should be some bill attachment option as it is very difficult to enter the data if the length of stay is more. And all data is added one by one. For	There should be a system for bulk upload in excel. We have a system of data entry in a single excel sheet regarding utilization of medicines so it is unnecessary duplication of the work. As I have said earlier to insert an import option for bulk excel sheets. Directly staff would go directly on the excel sheets whenever a patient is

			scanning the case and sending it. Also, medicines and lab if you are scanning like this it is easy. This is our suggestion.	each medicine, we have to fill 4 column and then we save it and add it and move to next and repeat the same process. This is very difficult. Ma'am we just want that either there is an attachment option so that we can attach the bills or they can be scanned. Or there should be a provision so that whatever is the total amount for let's say the medicines or investigations that can be added in the TMS.	discharged the staff would go on the excel and would directly link the same excel of the patient and patient gets discharged. Another issue is if patients is not discharged online and they seem on treatment from other hospitals so this is also an issue. So there should be a specific timeline for that and after which it should be automatically gets discharged. Pdf is easy to upload rather than entering the data. there would be a separate excel sheet where we would need to enter the details and later this excel sheet could uphanded with the patient record. One thing we can do is that the number of investigations that are being done the list we can get from, at the time of discharge we can get a sheet and uploading of that sheet will also work. Sheets upload, picture upload
			What I'm saying is, okay, this, the person system is, we have to improve the present information, present system, okay? And it should be informative. Okay. As my colleague told me earlier, it should be, you should be, it should be a little bit more informative because we are not get that much information with this present system.	Its important to have basic understanding why this was started because even we not aware why this was being done. Private sector will say this only. If it's not beneficial they can get de-empanel. But the people, and providers got the benefit. In DRG, only drugs and consumables are entered. You should also ask for our OT and IPD costs as they form a huge	I would have done that let me sync it first and after that it will be automatically copied in the other portal and then you fill in the data and again you don't need the same manpower. The person who is handling the TMS, doing the discharge, blocking the package and the other person would do the DRG thing so that is more convenient and time saving also. You cannot expect our team to work this as well as that. If we will do day wise data

				component of the costs incurred which should be included when package rates are set. Currently charges are bare minimum and we don't know what is the criteria for AB-PMAY	would be more feasible and accurate as well. The rate contracts are not there For example the patient is admitted today and the medicines are not available in our in-house pharmacy and we have MOU with Amrit pharmacy so that indent will go after approval from the MS office to the Amrit pharmacy and as we have to provide cashless services to the patients so those medicines or implants will be issued to the patients and the billing will be generated later on and their we cannot have costing data. ICD coding related. But yes, doctors don't write so it also hard for us to write.
			What I'm saying is, okay, this, the person system is, we have to improve the present information, present system, okay? And it should be informative. Okay. As my colleague told me earlier, it should be, you should be, it should be a little bit more informative because we are not get that much information with this present system.	Its important to have basic understanding why this was started because even we not aware why this was being done. Private sector will say this only. If its not beneficial they can get de-empanel. But the people, and providers got the benefit. In DRG, only drugs and consumables are entered. You should also ask for our OT and IPD costs as they form a huge component of the costs incurred which should be included when package rates are set. Currently charges are bare minimum and	

				we don’t know what is the criteria for AB-PMAY	
			1. It would be helpful if they get an additional training on the DRG, I mean ICD coding also. One more training. 2. they are suggesting that if they are able to get a video module training module, which they can refer to on how, which basically shows the step-by-step process of the data and train TMS. It would be very helpful for them to do this process. So that is one thing which, yes.	Online trainings were okay during Covid but there are so many issues. E.g., we don’t have electricity right now, so we had power cut for 3-4 hours every day since last week, how can we attend meetings online in this. Also, if we join from phone, we receive so many calls that it’s a headache if we don’t pick and if we pick we miss the trainings, I f we ask doubts, then they say we had cover it where were you. we need training because it’s difficult for day-to-day load of patients. The software also gets updated regularly and we have to figure it out by ourselves only. If every person involved is sensitized before involving, it will be good.	Either you give the whole responsibility of data entry to a clinical person and if you are giving it to a clinical person then it would hamper the patient care it has created a lot of disturbance. DRG sometimes work very slowly so cant it be just specifically for DRG and under which we can enter the patient OPD or patient Ayushman number and just after linking that we can upload. Can’t we even have a link system like we enter at one portal and that gets linked and gets updated from the same. We get the data from AMR and also we maintain the sheets so if somebody wants to come for an inspection that show of the details of a particular patient we can show you easily.
			DRG data entry is automatically generated from their HMIS system and even the patient's clinical documents, whatever is required at the claims part, that is also being generated through their HMIS system. And they're saying they have already communicated with the S H A and the NHL level asking for an		I think if this process in mandatory at the time of claim, we give time when patient process your claim.

			integration with the TMS, which would basically. You know, uh, make their life easier. Like all the data entry can be done. If they could, if the both the system could communicate with each other if there is such an integration. So that is one thing which they're mentioning because at their end, everything is generated now, uh, automated. Uh, it is automated, uh, at their end, but for the entry into our system, there is no other option other than doing it manually. they, if we could provide a provision wherein, they could upload the bills generated from their HMIS system, that would make their life easier. The other system, again, they are saying about the API integration between the hospital, HMIS, whatever is there, HMIS and the TMS system, wherein all this data, not just the DRG data, the patient's clinical data, whatever is being requested from the claim site and the other data and side of the PMJAY, all this is he, he's suggesting that at least 25 percentage of the work from the		

			hospital side would be reduced if such an integration is there.		
			I think if we can move that to the, you know, claim submission level where the claim is basically submitted to the processing team, if that is, that the mandatory documents which is submitted for the claims can be done at that point.	Most of the government institutions don't work online. If they want to do pilot, you should send their team, move desk to desk and collect all the data they need. They should either provide funds to us so that we can hire manpower or send a team on their payroll to collect all the data. They should supervise and monitor them. Otherwise, I don't think they will get the true results that they need.<br>Either someone is deputed for duty, who will fill DRG and we will do other file work. Provide proper funding for the staff recruited specifically for the collection of data from DRG. Those who are running in Ayushmann, they have only pooled the people. There should be a provision of hiring project specific person	So, 2 human resources should be there one and half where one for the data entry part and 50% of the time of a senior person who can do an audit or quality check to see whatever is entered is correct. But from our side we are done that if you don't provide us manpower, we are not going to provide the data. Like at national level it is reflected that Chattisgarh is doing well but at state level there should be a credibility for people who are working like Ayushman mitra's. Our accounting wing is working here they are billing everything they retrieve every data from each file and checks so that credit is also due and should be given to the workers
					created a different portal for entire study and that I would have synced with the TMS and this has no dealing with patient discharge
					category one surgical, one medical then taking sample and accurately entering the data is easy for us and

					then only it will be useful for your study. is it correct to collect the 100% data. If you would take less that would be good for us. Out of 100 cases if you put scientific sampling like 20% or 30% and you make a criteria like if you receive a patient of dialysis then DRG will be activated or not? I am not an IT person but I have understood your requirement that if a neurosurgery case comes to you, then DRG will be filled, if cardiac patient comes so, speciality wise you have defined that these many patients have come. If we have to do CTVS and we want the calculation of DVR that what will be the package cost of DVR then we will give you 2-3 samples to get the idea of the package from our side. 2. the batch changes the price also varies. In one batch the rate is this much and in another batch it will be different. So, you can't go for the uniform price.3. For finding these much PMAM we had to search a lot. Even for 1 manpower we have to fight a lot. This Account head is having so much work, she is overloaded. Either we get manpower/ technical support from them.
