# Supplementary material for: Establishing national hospital costing systems: insights from the qualitative assessment of cost surveillance pilot in Indian hospitals
Source: BMJ Open. 2024 Sep 10;14(9):e082965. doi: 10.1136/bmjopen-2023-082965 (PMC11409349; doi:10.1136/bmjopen-2023-082965)
Supplement: Supplementary file 4 [file bmjopen-2023-082965.reviewer_comments.pdf]

PEER REVIEW HISTORY

BMJ Open publishes all reviews undertaken for accepted manuscripts. Reviewers are asked to complete a checklist review form and are provided with free text boxes to elaborate on their assessment. These free text comments are reproduced below.

ARTICLE DETAILS

**TITLE (PROVISIONAL)**	Establishing National Hospital Costing Systems: Insights from the Qualitative Assessment of Cost Surveillance Pilot in Indian Hospitals
**AUTHORS**	Chugh, Yashika; Sharma, Shuchita; GUINNESS, LORNA; Sharma, Deepshikha; Garg, Basant; Mehndiratta, Abha; Prinja, Shankar

VERSION 1 - REVIEW

**REVIEWER NAME**	Hanssen, Odd Nikolas
**REVIEWER AFFILIATION**	United Nations Development Programme, Health Economics
**REVIEWER CONFLICT OF INTEREST**	I declare no competing interests.
**DATE REVIEW RETURNED**	12-Feb-2024

**GENERAL COMMENTS**	This paper looks to share the lessons learned from the experience of a pilot study of a national costing system, and is well-written, well-structured and thorough.<br>One major point to make is that the purpose or problem the paper seeks to address is not explicit and clear. It seems to be related to the difficulties in implementing a cost-reporting or cost-estimating system for health care delivery in an LMIC, but it never makes this statement directly.<br>The different sections could, thus, be made stronger (where relevant) by reflecting back on the 1.purpose of such costing systems, 2. the benefits of having such a system in place (its use) and most importantly 3. why it can be challenging to do so, in any LMIC context (rather than only specifically in this context).<br>It would also be good to understand if the costing system would be used to implement a different provider payment system. This is a major ramification if it is thought of as next step, and would bring the purpose of the study also to wider (and more specific) audiences.<br>Contentwise, the consideration of the usefulness of such systems, in terms of incentives of participating health care providers, would be the possibility to adjust to such provider payment systems, and the benefits (speed, accuracy of reimbursement, etc.) this may have.<br>In addition, it would be good to add some description of costing as a whole, noting that this exercise reflects on doing ingredients-based, bottom up costing to estimate the resources used during existing delivery of care, as this is just one of many applications of costing, and which determines a lot of the uses of its results, and the audiences that would be interested in this article.<br>Finally, if possible under characterization of interviewee responses, a quantitative representation of the issues in implementing the costing system would be powerful; the share (which seems high) of all respondents or facilities who noted no use of the costing system

	or significant issues with the training, etc., would be a better, visual way to present the prevalence of the implementation issues discussed.

**REVIEWER NAME**	Leeds, Ira
**REVIEWER AFFILIATION**	Yale School of Medicine
**REVIEWER CONFLICT OF INTEREST**	I have no competing interests
**DATE REVIEW RETURNED**	21-Feb-2024

**GENERAL COMMENTS**	The authors report a qualitative evaluation of efforts to developing costing systems in a novel public payer Indian healthcare system. Introduction of a public payer system with fair costing is important in the current development of social welfare programs in India, and the authors describe with a high degree of methodologic detail and breadth how these costing mechanisms were created barriers to implementation. The study seems very well done but the tone of the manuscript and the topic seem well outside the readership of a clinical journal like BMJ. This manuscript is essentially a technical paper for nonclinician healthcare administrators to model or follow when designing their own payment systems. There is very little that seems relevant for a clinician-oriented reader to gain from the findings described here.<br><br>For such an ambitious effort, it would also be helpful to have interviewed more than 21 respondents.<br><br>A minor concern is that the current format of 800-pages of appendices is quite overwhelming. It would be much more helpful if this information was included in a curated appendix that was both easier to read and easier to interpret.

**REVIEWER NAME**	Onatade, Raliat
**REVIEWER AFFILIATION**	King's College Hospital NHS Trust, Pharmacy Department
**REVIEWER CONFLICT OF INTEREST**	I have no competing interests.
**DATE REVIEW RETURNED**	12-Apr-2024

**GENERAL COMMENTS**	Thank you for this manuscript. Overall, I think that the paper would benefit greatly from a restructure of the results and discussion sections in particular. The background and aims gave a lot of initial promise which unfortunately was not borne out in the rest of the paper. The results section lacks some required detail. The discussion section could be made signficantly more focussed, giving a much shorter and more readable paper.<br>Please see some detailed comments below, I hope they are helpful.<br>Background- There appears to be some duplication of sentences.<br>Method - Better description of the participating sites in the study – hospital size, specialties, population served, digital maturity (e.g. relative use of manual vs paper systems for health records, prescribing etc.) is needed. Table 2 has some information, but not sufficient, e.g. a hospital with over 200 beds could be 210 beds, or 600 beds – therefore this classification does not allow for sufficient differentiation or understanding of the relative hospital sizes.<br>Please mention who conducted the interviews and their experience.

	More detail on how the tool was piloted is important.<br>The phrase 'google form circulated' is not sufficient description: more detail is needed please - for example - is this a 4th questionnaire or one of the three previously mentioned; elaboration on whether the form was a survey or questionnaire; how was it developed, validated and subsequently circulated; aimed at which participant groups.<br>Fig 2 – A priori themes - please consider if this information should form part of the results<br>Coding of transcripts – more detail is needed here – e.g. who did the coding, was it verified by a second person; how were disagreements resolved; did all the authors check the transcripts for coding accuracy or some of the authors (who should therefore be named)<br>Please consider adding a table for the details of the participants at each site. It is rather difficult to understand the information on participation otherwise.<br>Version of MS Excel used should be mentioned.<br>Results - Please follow the normal convention when using verbatim quotes from interviews, to aid the reader (quote, followed by an indication of who made the observation - data analyst/medical director and/or which site it originated from etc.), always being careful that the individual can't be identified<br>There is a sentence which says – 'x hospitals interviewed'. Hospitals cannot be interviewed, only people can be interviewed – I think these results need to be reported differently.<br>Please reconsider the use of the terms manpower and man-hours (except where these are direct quotes) and change to more inclusive language.<br>Some of the recommendations appear to include new information which might be better placed in the results section.<br><br>Discussion - First paragraph is describing what appear to be implementation problems. It is probably worth framing an element of the discussion aroud implementation failures, possibly using an existing framework. Use of a framework might also help in trying to provide some solutions.<br>It would be helpful to distinguish between implementation problems and difficulties caused by equipment and software deficiencies (e.g. slow portal)<br>A substantial part of the discussion lacks references for certain assertions, proposed solutions etc. This is despite there being a significant body of literature around coding, healthcare documentation and record keeping and healthcare data quality. Please add references to back up the assertions, or make clear where authors' expert opininon<br>The mention of countries which have done certain aspects of cost surveillance well is appreciated, but it is rather piecemeal, and it is not possible to understand where it is done well and success factors, in order to aid other countries. A more holistic and coherent comparison with cost-surveillance practices in other countries would be more helpful to point out where the current work could improve practice in India.

VERSION 1 – AUTHOR RESPONSE

Reviewer: 1

Dr. Odd Nikolas Hanssen, United Nations Development Programme

1. This paper looks to share the lessons learned from the experience of a pilot study of a national costing system, and is well-written, well-structured and thorough.

One major point to make is that the purpose or problem the paper seeks to address is not explicit and clear. It seems to be related to the difficulties in implementing a cost-reporting or cost-estimating system for health care delivery in an LMIC, but it never makes this statement directly. The different sections could, thus, be made stronger (where relevant) by reflecting back on the 1. purpose of such costing systems, 2. the benefits of having such a system in place (its use) and most importantly 3. why it can be challenging to do so, in any LMIC context (rather than only specifically in this context).

Response:

We are thankful to the reviewer for highlighting this aspect. We have now revised the Background section to explicitly state the aim of the study, highlight the purpose and benefits of routine cost-surveillance and the associated challenges with respect to LMICs. The revised text is as follows:

Page no. 6, Line no. 156-166:

"To ensure representativeness, these provider payment rates need to be updated regularly, which in turn is contingent on robust, timely and regularly available evidence on healthcare costs requiring routine collection of cost data.[12, 13] However, past experiences have shown that conducting large-scale costing studies can be extremely time and resource-intensive, especially in resource-constrained settings, and the evidence quickly becomes outdated.[12, 13] Additionally, the limited awareness and capacity regarding the use and benefits of healthcare cost accounting systems in lower and middle-income countries exacerbate the problem.[14, 15] Therefore, it is crucial to build sustainable systems that can provide routine estimates with minimal additional effort from healthcare providers."

Page no. 6, Line no. 193-200:

"This study aims to identify the challenges in implementing such a cost-surveillance system for its long-term sustainability. To document and learn from the initial difficulties of this data collection effort, we conducted a qualitative process evaluation of the hospital cost reporting pilot. We aim to describe the processes followed and the challenges faced by both providers as well as the payer agency. Secondly, we provide recommendations for setting-up such a system in India as well as other lower- and middle-income countries (LMICs)."

2. It would also be good to understand if the costing system would be used to implement a different provider payment system. This is a major ramification if it is thought of as next step, and would bring the purpose of the study also to wider (and more specific) audiences.

Response:

We appreciate the reviewer's insightful comment regarding the potential implementation of a different provider payment system. We would like to mention that the cost-surveillance pilot was undertaken with the long-term aim of exploring the possibility to eventually transition to Diagnosis-Related Group (DRG) based provider payment system. However, the explicit purpose of this pilot was to establish a cost-surveillance system that enables the routine collection of patient-level resource utilization data.

As part of the broader payment reforms, several other consultations were also being simultaneously conducted to evaluate the suitability of the DRG payment systems. However, since the objective of this paper is to assess the feasibility and effectiveness of the cost-surveillance system specifically, we have not described this long-term policy objectives in detail. Moreover, the broader policy objective was still under consideration and not a committed strategy with a clear implementation plan at the time of the study.

In light of the reviewer's comment, we have added a line to provide context on the long-term objective (page no. 8, line no. 205-208): " This process evaluation of the cost-surveillance pilot will also guide the feasibility and potential application of a diagnostic related groups (DRG)-based provider payment system in the future."

3. Content-wise, the consideration of the usefulness of such systems, in terms of incentives of participating health care providers, would be the possibility to adjust to such provider payment systems, and the benefits (speed, accuracy of reimbursement, etc.) this may have.

Response:

We agree with the reviewer that the long-term motivation for hospitals to participate in the pilot is to transform to the revised provider payment system which may better reflect the costs and is more responsive. However, providing monetary or non-monetary incentives can be other options to elicit participation of hospitals. In the current cost-surveillance pilot activity there was no additional monetary or non-monetary incentive for undertaking this additional work. However, possibility of exploring some incentive structures would be necessary to evaluate. We have mentioned the same in our manuscript in the “way forward” section.

Page no. 18, line no. 535-538

A potential way to improve the system is to motivate the providers by incentivising them to provide quality information or by prioritizing their claim processing. Providing such preferential system will not entail any additional financial liability on the payer.

4. In addition, it would be good to add some description of costing as a whole, noting that this exercise reflects on doing ingredients-based, bottom-up costing to estimate the resources used during existing delivery of care, as this is just one of many applications of costing, and which determines a lot of the uses of its results, and the audiences that would be interested in this article.

Response:

We are thankful to the reviewer for highlighting this aspect. We have now revised the Background section to provide details on the costing approach utilised as well as the necessary references for greater detail. The revised text is as follows:

Page no. 5-6, Line no. 150-155:

“The estimation of these healthcare delivery costs employed a mix of top-down and bottom-up micro-costing approaches. The top-down method uses retrospective healthcare expenditure data, dividing total costs by the services provided within a specific period, offering a broad expense overview. The bottom-up approach disaggregates data to detail each resource used, providing higher accuracy but being more time-consuming.[10, 11]”

5. Finally, if possible, under characterization of interviewee responses, a quantitative representation of the issues in implementing the costing system would be powerful; the share (which seems high) of all respondents or facilities who noted no use of the costing system or significant issues with the training, etc., would be a better, visual way to present the prevalence of the implementation issues discussed.

Response:

We thank the reviewer for this useful suggestion. While we quantify the difficulties to some extent by reporting the challenges related capturing the details on drugs and consumables in Figure 5, we have now added an additional table to summarise the issues in terms of what percentage of providers faced a certain challenge including the lack of motivation, additional workload, shortage of manpower, limited training and the issues related to the transaction management system. The following table has been added to the text:

Table 5b: Challenges face by healthcare providers while reporting cost-surveillance data

S. No. Challenges faced by healthcare providers (N=21) n (%)

1 Lack of motivation to participate 17 (80.95%)

2 Increased workload & shortage of manpower 15 (71.43%)

3 Limited competency of manpower to comprehend medical information 16 (76.19%)

4 Inadequacy of the training process 7 (33.33%)

5 Issues with the transaction management system 11 (52.38%)

Reviewer 2:

Dr. Ira Leeds, Yale School of Medicine

1. The authors report a qualitative evaluation of efforts to developing costing systems in a novel public payer Indian healthcare system. Introduction of a public payer system with fair costing is important in the current development of social welfare programs in India, and the authors describe with a high degree of methodologic detail and breadth how these costing mechanisms were created barriers to implementation. The study seems very well done but the tone of the manuscript and the topic seem well outside the readership of a clinical journal like BMJ. This manuscript is essentially a technical paper for non-clinician healthcare administrators to model or follow when designing their own payment systems. There is very little that seems relevant for a clinician-oriented reader to gain from the findings described here.

Response:

We appreciate your feedback on our manuscript and acknowledge your concerns regarding its relevance to a clinical journal like BMJ. However, we would like to emphasize that BMJ encompasses a broad spectrum of collections, including those focused on Health Policy, Health Services Research, and Health Economics. Our manuscript aligns well with these areas, integral to the comprehensive understanding and improvement of healthcare systems. The topic of establishing fair and accurate costing systems in public healthcare is critical for the sustainability and efficiency of healthcare systems, particularly in low- and middle-income countries. The lessons learned from our study can

provide a valuable framework for similar initiatives worldwide, underscoring the importance of this research in the global health context.

We also wish to draw attention to several related articles previously published in BMJ collections, which discuss health policy, health economics, and services research, further supporting the relevance of our manuscript to BMJ's readership:

https://doi.org/10.1136/bmjopen-2023-078390

https://doi.org/10.1136/bmjopen-2023-076853

https://doi.org/10.1136/bmjopen-2023-073913

https://doi.org/10.1136/bmjopen-2023-074341

https://doi.org/10.1136/bmjopen-2023-075030

https://doi.org/10.1136/bmjopen-2022-065019

We believe that the insights provided by our research will be beneficial not only to healthcare administrators but also to clinicians who are increasingly involved in healthcare management and policy decisions.

2. For such an ambitious effort, it would also be helpful to have interviewed more than 21 respondents.

Response:

We appreciate the reviewer’s comment regarding the sample size limitation. However, we would like to highlight that interviews were conducted across 21 hospitals, with 4-5 respondents per hospital. These respondents included medical superintendents, medical directors, data entry operators, administrative in-charges, hospital owners, and accounts and finance officers. To ensure representativeness, we selected a diverse mix of hospitals based on ownership, bed capacity, enrolment status in the hospital cost surveillance pilot, and accreditation status by the National Accreditation Board for Hospitals & Healthcare Providers (NABH).

Additionally, to strengthen our findings, we organized a panel discussion with 32 experts representing a wide array of stakeholders. These included government agencies, non-governmental organizations, healthcare provider associations, the private healthcare sector, industry partners, and health insurance representatives. The healthcare provider associations covered a variety of entities within India’s healthcare landscape, such as hospitals, nursing homes, clinics, diagnostic centres, medical equipment companies, and insurance providers. This ensured a comprehensive representation of India's healthcare ecosystem, incorporating diverse viewpoints. The outcomes of the panel discussion were subsequently shared with participants for their review and feedback, which were then integrated into the manuscript.

Nonetheless, we understand the reviewer's apprehension regarding the limited sample size. Hence, we have acknowledged this as a limitation of the study under the Strengths and Limitations Section at Page no. 5, line no. 93-100:

• “A comprehensive process evaluation of the healthcare cost surveillance pilot from the perspective of both providers and payer agencies.

• The characteristics of the providers interviewed included public, private, and trust hospitals with various bed capacities and accreditations by the National Accreditation Board for Hospitals & Healthcare Providers (NABH) to ensure representativeness.

• The interviews were limited to three states, potentially missing broader national perspectives.

• Virtual interviews in Kerala versus face-to-face interviews in Haryana and Chhattisgarh may introduce variability in responses"

3. A minor concern is that the current format of 800-pages of appendices is quite overwhelming. It would be much more helpful if this information was included in a curated appendix that was both easier to read and easier to interpret.

Response:

We thank the reviewer for highlighting this. Given the suggestion, we have now revised the format of the file from MS Excel to Word for the ease of the reader. The revised annexure (Annexure 2) has been submitted with the revision.

Reviewer 3:

Dr. Raliat Onatade, King's College Hospital NHS Trust, King's College London

1. Thank you for this manuscript. Overall, I think that the paper would benefit greatly from a restructure of the results and discussion sections in particular. The background and aims gave a lot of initial promise which unfortunately was not borne out in the rest of the paper. The results section lacks some required detail. The discussion section could be made significantly more focussed, giving a much shorter and more readable paper. Please see some detailed comments below, I hope they are helpful.

Response:

We are thankful for the insightful comments on our manuscript. We appreciate your thorough review and constructive feedback. We have carefully considered your suggestions and made significant revisions to address the concerns raised. Specifically, we have restructured the background sections to ensure alignment with the study objectives and the findings reported. Additionally, we have also restructured the discussion section, employing an implementation framework to classify the challenges and propose relevant solutions aimed to make the paper more reader-friendly and concise while still addressing the key points raised in the study. We have also addressed all other comments and suggestions you provided, providing point-by-point responses for each below.

2. Background- There appears to be some duplication of sentences.

Response:

We thank the reviewer for the suggestion. We have now revised the background section to delete duplicate sentences.

3. Method - Better description of the participating sites in the study – hospital size, specialties, population served, digital maturity (e.g. relative use of manual vs paper systems for health records, prescribing etc.) is needed. Table 2 has some information, but not sufficient, e.g. a hospital with over 200 beds could be 210 beds, or 600 beds – therefore this classification does not allow for sufficient differentiation or understanding of the relative hospital sizes.

Response:

We have now revised table 1 to include details on hospital size, specialities served as well as digital maturity.

4. Please mention who conducted the interviews and their experience.

Response:

The interviews were conducted by Yashika Chugh (YC) and Shuchita Sharma (SS). Both of them have significant experience in qualitative research in healthcare. They have conducted numerous interviews and focus group discussions in healthcare settings. Both YC and SS hold Master's degree in Public Health with an experience of 5 and 4 years respectively. Both of them have been working around generating evidence and support the rationalisation for PM-JAY health benefit packages.

5. More detail on how the tool was piloted is important.

Response:

We thank the reviewer for raising this important point. We have now revised the text to add details on the pilot process. The text has been revised as follows:

Page no. 9, Line no. 252-258:

"The tool was developed collaboratively by SS, YC, and DS. To ensure its validity and reliability in gathering the necessary information, it was reviewed and revised multiple times by SP and LG. A pilot was conducted by administering the tool to three health facilities: a private tertiary hospital, a government tertiary hospital, and a government district hospital. Feedback was incorporated at each stage to refine the questions for clarity and ease of understanding. The pilot phase concluded when no significant feedback or difficulties in comprehension were reported by the providers."

6. The phrase 'google form circulated' is not sufficient description: more detail is needed please - for example - is this a 4th questionnaire or one of the three previously mentioned; elaboration on whether the form was a survey or questionnaire; how was it developed, validated and subsequently circulated; aimed at which participant groups.

Response:

We appreciate the reviewer's concern. We would like to clarify that at the end of each interview, participants were provided with a survey to complete and submit later. This survey was specifically designed for healthcare providers and focused on the difficulty level of cost reporting activities based on the type of information being reported. Under the cost-surveillance pilot, patient-level data on quantity and price of resources consumed, i.e., drugs, consumables, implants, and diagnostics were being collected. The survey was therefore designed using a Likert scale to assess the difficulty level

for each of these. The Annexure I has now been revised to include this part as well. The findings from this survey are presented in the results section as Figure 5b. The following changes have been made to the text:

Page no. 10, Line no. 270-274:

"In addition, a survey was also conducted amongst the healthcare providers which was aimed to capture the difficulty level in cost reporting activity according to the type information being reported, i.e., for quantity and price of resources consumed including drugs, consumables, implants, and diagnostics."

7. Fig 2 – A priori themes - please consider if this information should form part of the results

Response:

We are thankful to the reviewer for this suggestion. We would like to mention that a priori themes are typically identified through the interview tool before the interviews to ensure all necessary information is captured. Therefore, we initially placed them in the methods section. However, we understand the reviewer's perspective and have moved the figure to the results section to better align with the presentation of our findings.

8. Coding of transcripts – more detail is needed here – e.g. who did the coding, was it verified by a second person; how were disagreements resolved; did all the authors check the transcripts for coding accuracy or some of the authors (who should therefore be named)

Response:

We have now added the necessary details as suggested by the reviewer. The following changes have been made in the text:

Page no. 10, Line no. 279-281:

"Microsoft Excel version 2019 was used to categorise and code the data gathered from all the interviews. Coding was done by SS and YC and in case of any disagreement, it was resolved by LG and SP. All the transcripts for were checked for accuracy by LG, DS and SP."

9. Please consider adding a table for the details of the participants at each site. It is rather difficult to understand the information on participation otherwise.

Response:

We have now added a table (2) to describe the participant characteristics (medical superintendent/medical directors/data entry operators and Pradhan Mantri Ayushman Mitra (PMAM)/administrative in-charge/ hospitals owners/accounts/finance officers) at each study site.

10. Version of MS Excel used should be mentioned.

Response:

We have now added the necessary details as suggested by the reviewer. The following changes have been made in the text:

Page no. 10, Line no. 279-280:

“Microsoft Excel version 2019 was used to categorise and code the data gathered from all the interviews.”

11. Results - Please follow the normal convention when using verbatim quotes from interviews, to aid the reader (quote, followed by an indication of who made the observation - data analyst/medical director and/or which site it originated from etc.), always being careful that the individual can't be identified.

Response:

We thank reviewer for this suggestion. We have now indicated who made the observation and the site as well. However, we would like to mention that not all quotes have been placed in the text due to issue of the word count. The table of quotes has been attached as Annexure 2.

12. There is a sentence which says – 'x hospitals interviewed'. Hospitals cannot be interviewed, only people can be interviewed – I think these results need to be reported differently.

Response:

We have now revised this sentence as follows:

Page no.12, line no. 348-50

“Further, respondents from more than half of the hospitals (16 out of 21) interviewed highlighted that the online training sessions were mostly attended by the higher staff.”

13. Please reconsider the use of the terms manpower and man-hours (except where these are direct quotes) and change to more inclusive language.

Some of the recommendations appear to include new information which might be better placed in the results section.

Response:

We have now revised the terms as “manpower” to “human resources” to make it more inclusive. In addition, we would like to mention that “Recommendations” was one the themes under our results section and these recommendations were provided by the respondents interviewed.

The following changes have been made in the text:

Page no.14, line no. 397-399

“This means that in high volume centres, there would be an average of 30 (19 to 65) additional person-hours required per day per hospital.”

14. Discussion - First paragraph is describing what appear to be implementation problems. It is probably worth framing an element of the discussion around implementation failures, possibly using an existing framework. Use of a framework might also help in trying to provide some solutions. It would be helpful to distinguish between implementation problems and difficulties caused by equipment and software deficiencies (e.g. slow portal)

Response:

We thank the reviewer for this suggestion. We would like to mention that the first paragraph of the discussion summarises the key findings from the results and not only implementation problems. As suggested by the reviewer, we have tried to re-structure the discussion using an implementation framework which includes discussion on criteria including acceptability, fidelity and feasibility of any health intervention or program, ultimately, guiding its sustainability and scalability. The following changes have been made to the text:

Page no. 11, Line no. 303-305:

"To better understand the challenges, findings are discussed, using an implementation science framework considering acceptability, fidelity, and feasibility—factors that ultimately guide the sustainability and scalability of an intervention or program."

Page no. 17, Line no. 509-513:

In terms of acceptability, preliminary findings indicate that the current approach places a significant burden on healthcare providers, leading to hesitance and unease in participation. This can potentially compromise the quality of collected data and affect decision-making in the healthcare sector. The success of provider payment reforms relies heavily on high-quality data."

Page no. 18, Line no. 514-520:

"While assessing fidelity, which implies evaluating adherence to program requirements, we identified critical factors ensuring data accuracy, despite not having access to the pilot's collected data. The design of a case-based payment system hinges on the quality of patient records, which depends on accurately distinguishing between different diagnoses and procedures and patient complexity within a single group. The adequacy of clinical documentation, availability of skilled staff, and consistent training influence the accuracy of this information."

Page no. 19, Line no. 544-555:

"Thirdly, the findings highlight several caveats in the training process which is key to ensure accuracy of the data being collected, especially given the limited qualification of data entry staff. Bridging the gap between training and implementation is crucial, directly imparting knowledge to those involved. Tailored training sessions, supplemented with supportive supervision, will enhance proficiency. More importantly, regular monitoring, feedback, and guidance to address challenges are pivotal for continuous improvement.

The third key aspect guiding the sustainability and scalability of a program is its feasibility. Our study reveals that the cost surveillance data entry process, particularly concerning consumables, is notably time-consuming. National costing studies in India have shown that consumables, along with human resources and drugs, constitute a significant proportion of the total cost."

15. A substantial part of the discussion lacks references for certain assertions, proposed solutions etc. This is despite there being a significant body of literature around coding, healthcare documentation and record keeping and healthcare data quality. Please add references to back up the assertions, or make clear where authors' expert opinions.

Response:

We are thankful to the reviewer for raising this concern. We agree with the reviewer and have now revised the text to add references wherever necessary. The following references have been added (23-42):

“23. Klaic M, Kapp S, Hudson P, Chapman W, Denehy L, Story D, Francis JJ. Implementability of healthcare interventions: an overview of reviews and development of a conceptual framework. Implementation Science. 2022 Jan 27;17(1):10.

24. Ndayishimiye C, Tambor M, Dubas-Jakóbczyk K. Barriers and Facilitators to Health-Care Provider Payment Reform–A Scoping Literature Review. Risk Management and Healthcare Policy. 2023 Dec 31:1755-79.

25. Bredenkamp C, Bales S, Kahur K, editors. Transition to Diagnosis-Related Group (DRG) payments for health: lessons from case studies. World Bank Publications; 2019 Dec 19.

26. Barber SL, Lorenzoni L, Ong P. Institutions for health care price setting and regulation: A comparative review of eight settings. The International Journal of Health Planning and Management. 2020 Mar;35(2):639-48.

27. World Health Organization. Case-based Payment Systems for Hospital Funding in Asia An Investigation of Current Status and Future Directions: An Investigation of Current Status and Future Directions.

28. Mathauer I, Wittenbecher F, World Health Organization. DRG-based payments systems in low- and middle-income countries: Implementation experiences and challenges. World Health Organization; 2012.

29. Tan SS, Geissler A, Serdén L, Heurgren M, Van Ineveld BM, Redekop WK, Hakkaart-van Roijen L. DRG systems in Europe: variations in cost accounting systems among 12 countries. The European Journal of Public Health. 2014 Dec 1;24(6):1023-8.

30. Vogl M. Assessing DRG cost accounting with respect to resource allocation and tariff calculation: the case of Germany. Health economics review. 2012 Dec;2(1):1-2.

31. Haigekassa E. Overview of Estonian experiences with DRG system. Tallin, Estonia: Department of Health Economics, Estonian Health Insurance Fund. 2009.

32. Berki SE. The design of case-based hospital payment systems. Medical Care. 1983 Jan 1;21(1):1-3.

33. Campbell S, Giadresco K. Computer-assisted clinical coding: A narrative review of the literature on its benefits, limitations, implementation and impact on clinical coding professionals. Health Information Management Journal. 2020 Jan;49(1):5-18.

34. Tripathy JP, Bahuguna P, Prinja S. Drug prescription behavior: A cross-sectional study in public health facilities in two states of North India. Perspectives in clinical research. 2018 Apr 1;9(2):76-82.

35. Mishra R, Harsvardhan R, Rai R, Chandra H. A study to assess the degree of adherence of prescription to WHO and MCI guidelines at a tertiary health care teaching hospital in North India. Journal of Patient Safety and Infection Control. 2019 May 1;7(2):48-52.

36. Leventhal R (2014) Recognizing the value of clinical documentation improvement. Healthcare Informatics [online]. Available at: https://www.healthcare-informatics.com/article/recognizing-value-clinical-documentation-improvement (accessed 05 June 2024).

37. Towers A (2013) Clinical documentation improvement – A physician perspective: Insider tips for getting physician participation in CDI programs. Journal of AHIMA 84(7): 34–41.

38. Hay P, Wilton K, Barker J, Mortley J, Cumerlato M. The importance of clinical documentation improvement for Australian hospitals. Health Information Management Journal. 2020 Jan;49(1):69-73.

39. Wilson LN. The Impact of a Clinical Documentation Improvement Program (Doctoral dissertation).

40. McGinn, C.A., Grenier, S., Duplantie, J., Shaw, N., Sicotte, C., Mathieu, L., Leduc, Y., Légaré, F. & Gagnon, M.-P. 2011. Comparison of user groups' perspectives of barriers and facilitators to implementing electronic health records: a systematic review. BMC medicine, 9(1):46.

41. Senne GL. Exploring challenges in records management in a public hospital setting (Doctoral dissertation, North-West University (South Africa)).

42. Syzdykova A, Malta A, Zolfo M, Diro E, Oliveira JL. Open-source electronic health record systems for low-resource settings: systematic review. JMIR medical informatics. 2017 Nov 13;5(4):e8131."

16. The mention of countries which have done certain aspects of cost surveillance well is appreciated, but it is rather piecemeal, and it is not possible to understand where it is done well and success factors, in order to aid other countries. A more holistic and coherent comparison with cost-surveillance practices in other countries would be more helpful to point out where the current work could improve practice in India.

Response:

We Understand the reviewer's concern. We would like to mention that the country examples quoted are reflective of successful practices and their experience over the journey to develop strong cost-surveillance systems. In addition, a detailed comparison of these countries on the key features of their cost surveillance system and its evolution has been provided as Annexure 3 (with references).

VERSION 2 - REVIEW

**REVIEWER NAME**	Hanssen, Odd Nikolas
**REVIEWER AFFILIATION**	United Nations Development Programme, Health Economics
**REVIEWER CONFLICT OF INTEREST**	I declare no competing interests
**DATE REVIEW RETURNED**	04-Jul-2024

**GENERAL COMMENTS**	This article as written now is more accurate in what it looks to assess and contribute to the literature, namely the process of doing a costing study in an LMIC as part of setting better reimbursement

	rates based on the actual costs and resource requirements the system needs to deliver care.<br>There may be some debate on whether some claims made regarding what would be better for the system (disaggregation of cases based on severity and co-morbidity) or how and why the current reimbursement formula differs from DRGs, but these are not central to the purpose of the paper. A choice could be made to avoid any discussion of this to avoid distraction on the part of the reader, but this is a subjective choice.<br>Minor improvements to the writing of the paper are still required (f ex. the use of assurance over insurance, lack of 'the' when needed, etc.), and there are times that the paper is repetitive, stating the same things, almost to being the exact same text, in multiple places. This should not relatively straightforward to address as well.<br>If anything, if the authors want to incorporate something else, the mention of variation within the country on costs was very interesting, and in parallel, and more central to the study, any particular findings that reflect specific differences between the study sites could merit discussion, such as reflecting on more pronounced differences in the challenges presented as a result of local differences (certain regions have greater HR/limitations because of less overall resources and capacity constraints, which mean these are less able to be part of the costing study and deliver cost data or eventually adopt this or a DRG system, for example).<br>Otherwise, generally, a solid paper.

VERSION 2 – AUTHOR RESPONSE

Reviewer 1:

Dr. Odd Nikolas Hanssen, United Nations Development Programme

1. This article as written now is more accurate in what it looks to assess and contribute to the literature, namely the process of doing a costing study in an LMIC as part of setting better reimbursement rates based on the actual costs and resource requirements the system needs to deliver care. There may be some debate on whether some claims made regarding what would be better for the system (disaggregation of cases based on severity and co-morbidity) or how and why the current reimbursement formula differs from DRGs, but these are not central to the purpose of the paper. A choice could be made to avoid any discussion of this to avoid distraction on the part of the reader, but this is a subjective choice.

Response:

We thank the reviewer for the suggestion. We would like to mention that we introduced the concept of DRGs as a potential future provider payment system to illustrate the rationale behind this study's process evaluation of the pilot, as recommended by the reviewer in the previous revision. We do not intend to advocate for a specific reimbursement model, whether a flat rate, or sensitive to patient characteristics, or a full migration to DRGs for the Indian context. The discussion around these payment systems was included solely to provide a rationale for the current analysis and not to make any definitive claims about their suitability.

2. Minor improvements to the writing of the paper are still required (f ex. the use of assurance over insurance, lack of 'the' when needed, etc.), and there are times that the paper is repetitive, stating the

same things, almost to being the exact same text, in multiple places. This should be relatively straightforward to address as well.

Response:

We appreciate the feedback on the language and structure of the paper. We have reviewed the manuscript and made the necessary corrections.

Regarding the use of the term "assurance" for PM-JAY, we use it instead of "insurance" to emphasize the program's focus on providing guaranteed healthcare access and financial protection to beneficiaries. Unlike traditional insurance, which typically involves conditional benefits based on risk assessments, PM-JAY operates more as a social assurance mechanism, ensuring that eligible individuals receive healthcare services without financial barriers. However, as suggested by the reviewer, we have revised the text to use the term “insurance”.

3. If anything, if the authors want to incorporate something else, the mention of variation within the country on costs was very interesting, and in parallel, and more central to the study, any particular findings that reflect specific differences between the study sites could merit discussion, such as reflecting on more pronounced differences in the challenges presented as a result of local differences (certain regions have greater HR/limitations because of less overall resources and capacity constraints, which mean these are less able to be part of the costing study and deliver cost data or eventually adopt this or a DRG system, for example). Otherwise, generally, a solid paper.

Response:

We thank the reviewer for this suggestion. We would like to mention that the primary objective of this paper was to conduct a process evaluation of the ongoing cost-surveillance activity, rather than to evaluate the any supply side or other differences across regions. As such, no additional data was collected specifically to analyze these variations. However, we acknowledge the heterogeneity within the country and the potential impact of local differences on healthcare delivery. Table 1 in the manuscript provides some context of characteristics of the facilities in the different study sites including details on the hospital size, specialties served, and digital maturity of the study sites. This information helps illustrate the diversity of the sites involved in the study and provides a background to understand the broader context of the process evaluation.
